# Copy Number Variation in Intron 1 of *SOX5* Causes the Pea-comb Phenotype in Chickens

**DOI:** 10.1371/journal.pgen.1000512

**Published:** 2009-06-12

**Authors:** Dominic Wright, Henrik Boije, Jennifer R. S. Meadows, Bertrand Bed'hom, David Gourichon, Agathe Vieaud, Michèle Tixier-Boichard, Carl-Johan Rubin, Freyja Imsland, Finn Hallböök, Leif Andersson

**Affiliations:** 1 Department of Medical Biochemistry and Microbiology, Uppsala University, Uppsala, Sweden; 2 Department of Neuroscience, Uppsala University, Uppsala, Sweden; 3 INRA, AgroParisTech, UMR1313 Animal Genetics and Integrative Biology, Jouy-en-Josas, France; 4 INRA, UE1295 PEAT, Nouzilly, France; 5 Department of Medical Sciences, Uppsala University Hospital, Uppsala, Sweden; 6 Department of Animal Breeding and Genetics, Swedish University of Agricultural Sciences, Uppsala, Sweden; Princeton University, Howard Hughes Medical Institute, United States of America

## Abstract

*Pea-comb* is a dominant mutation in chickens that drastically reduces the size of the comb and wattles. It is an adaptive trait in cold climates as it reduces heat loss and makes the chicken less susceptible to frost lesions. Here we report that *Pea-comb* is caused by a massive amplification of a duplicated sequence located near evolutionary conserved non-coding sequences in intron 1 of the gene encoding the SOX5 transcription factor. This must be the causative mutation since all other polymorphisms associated with the *Pea-comb* allele were excluded by genetic analysis. *SOX5* controls cell fate and differentiation and is essential for skeletal development, chondrocyte differentiation, and extracellular matrix production. Immunostaining in early embryos demonstrated that *Pea-comb* is associated with ectopic expression of *SOX5* in mesenchymal cells located just beneath the surface ectoderm where the comb and wattles will subsequently develop. The results imply that the duplication expansion interferes with the regulation of *SOX5* expression during the differentiation of cells crucial for the development of comb and wattles. The study provides novel insight into the nature of mutations that contribute to phenotypic evolution and is the first description of a spontaneous and fully viable mutation in this developmentally important gene.

## Author Summary

The featherless comb and wattles are defining features of the chicken. Whilst the *Pea-comb* allele was known to show a dominant inheritance and drastically reduce the size of both comb and wattles, the genetics underlying the mutation remained elusive. Chicken comb is primarily composed of collagen and hyaluronan, which are produced by chondrocytes. These cells are formed through the condensation and differentiation of mesenchyme cells during the chondrogenesis pathway, the early stages of which are regulated by *SOX* transcription factors. Here we pinpoint a massive amplification of a duplicated sequence in the first intron of *SOX5* as causing the Pea-comb phenotype. By studying early embryos, we show that SOX5 is ectopically expressed during a restricted stage of development in the cells which underlie the comb and wattles of Pea-comb animals. We hypothesise that the sequence duplication alters the regulation of SOX5 expression when the differentiation of cells essential for comb and wattle development is taking place. Pea-comb adds to the growing list of phenotypic variation which is explained by regulatory mutations and so demonstrates the evolutionary significance of such events.

## Introduction

**Figure 1:**
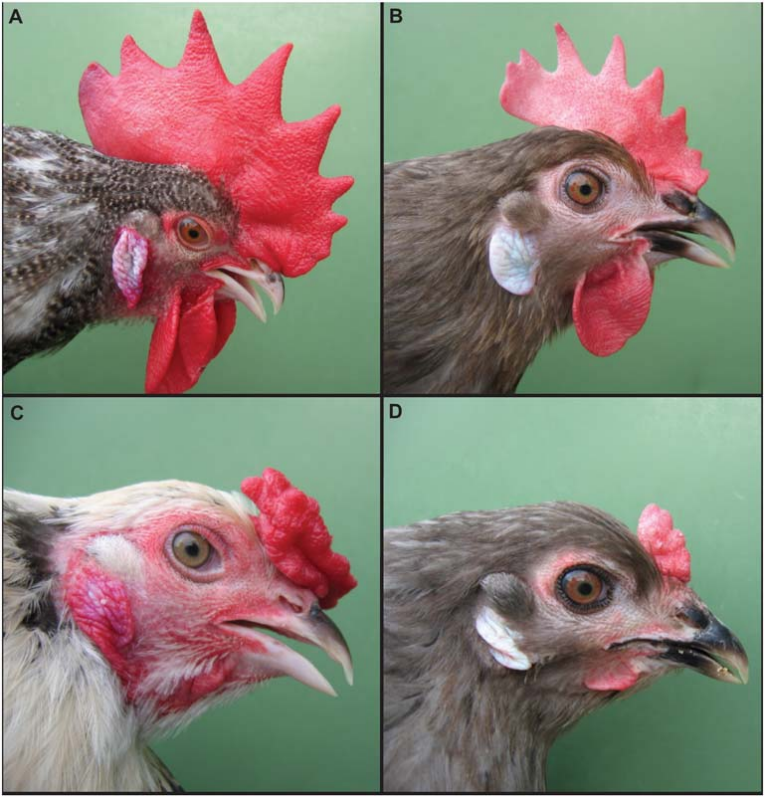
Wild-type and Pea-comb chickens. (A) Wild-type male, (B) wild-type female, (C) Pea-comb male and (D) Pea-comb female (Photo by David Gourichon).

In 1902 Bateson [1] reported the first examples of Mendelian inheritance in animals based on the genetic studies of four traits in chicken, one of these being the Pea-comb phenotype (Figure 1). The *Pea-comb* allele results in reduced comb and wattle size compared to wild-type individuals. *Pea-comb* shows incomplete dominance and as such the small comb shape can differ slightly between homo- and heterozygous birds. Homozygotes present three longitudinal rows of papillae, whilst heterozygotes can have a well-developed central blade (still of reduced size compared to wild-type) [2]. The wild-type has a single central blade of tissue and is therefore often denoted single comb. Bateson and Punnet [3] reported the first example of an epistatic interaction between genes when they showed that walnut comb is caused by the combined effect of *Pea-comb* and *Rose-comb*. Subsequent studies revealed that *Pea-comb*, besides its effect on comb and wattles, was also associated with a ridge of thickened skin that runs the length of the keel over the breast bone [4]. The *Pea-comb* mutation may have occurred early during domestication as the phenotype is widespread among both European and Asian breeds of chickens. Furthermore, it has been speculated that a reproduction in the tomb of Rekhmara at Thebes, Egypt, dated to ∼3,450 years before present depicts a rooster with the characteristic Pea-comb phenotype [5].

Chickens were domesticated from the red junglefowl with some contributions from the grey junglefowl [6], two species adapted to subtropical or tropical environments. Chickens do not sweat, instead they dissipate up to 15 percent of their body heat through the comb and wattles [7], making the Pea-comb phenotype adaptive to cold environments since it reduces heat loss. This phenotype has also been favoured in chickens bred for cock-fighting, as noted by Darwin [8] the smaller ornaments provided smaller targets for injury.

In the present study we show that the classical Pea-comb phenotype in chickens is caused by a large expansion of a duplicated sequence in intron 1 of the gene for the SOX5 transcription factor.

## Results

### Identifying the Causative Gene for Pea-comb

**Figure 2:**
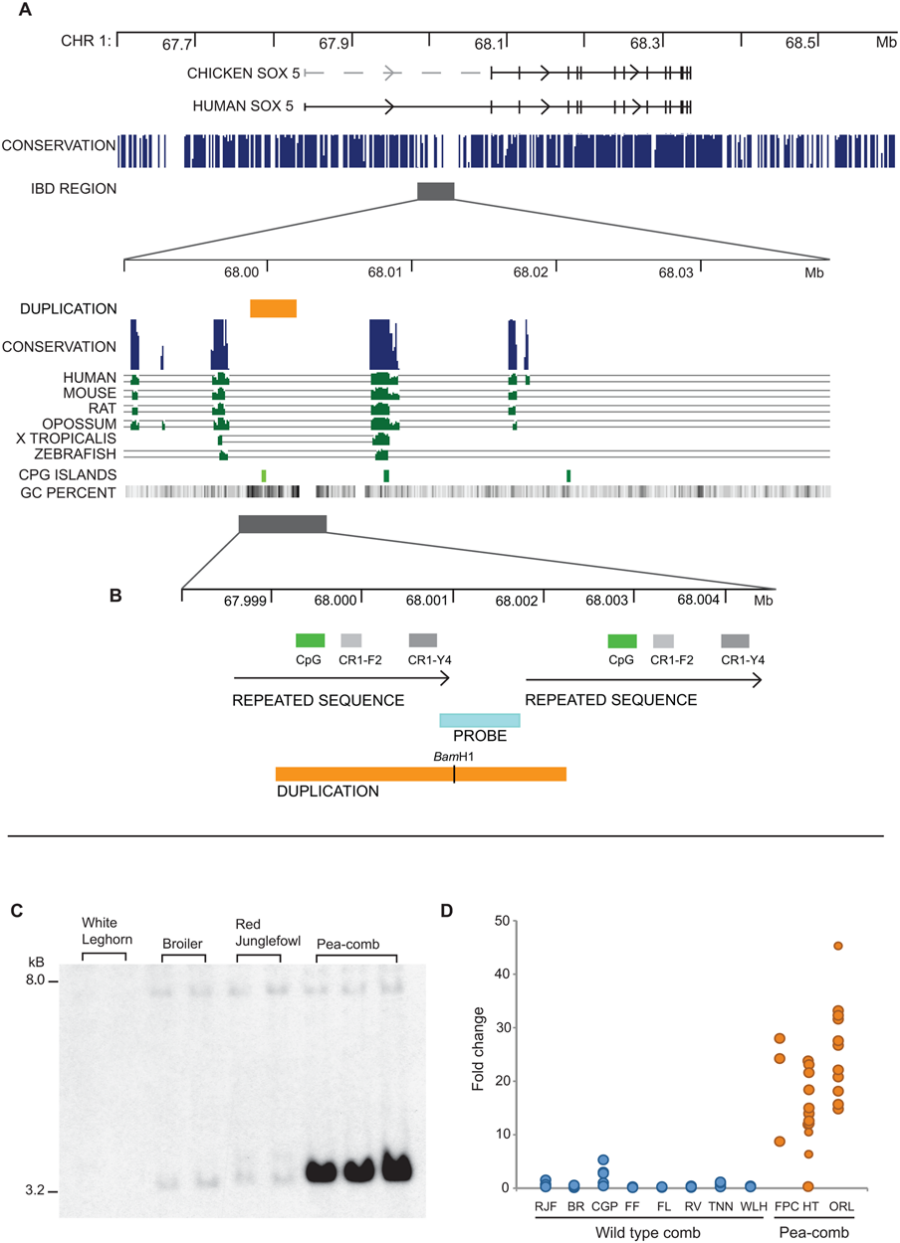
Identification of the *Pea-comb* mutation. (A) The region on chicken chromosome 1 harbouring *SOX5*. The conservation score track shows the large number of Evolutionary Conserved Non-Coding Elements at the *SOX5* locus. The region upstream of *SOX5* exon 2 identified through IBD mapping and showing complete association to *Pea-comb* is marked with a dark shaded bar. Bottom part: the *Pea-comb* IBD region is expanded. The position of the 3.2 kb duplicated sequence in the near vicinity of non-coding sequences conserved across vertebrate species is marked with an orange bar (adapted from the UCSC genome browser http://genome.ucsc.edu/). The GC content and the location of CpG islands are indicated. (B) Localization and composition of the duplicated sequence. CR1-F2 and CR1-Y4 are partial LINEs and a small CpG island is marked with a green bar. The region corresponding to the probe used for Southern blot analysis is indicated. (C) Southern blot analysis using genomic DNA digested with *Bam*HI from Pea-comb and wild-type chickens; the estimated sizes of restriction fragments are given to the left. (D) Results of real-time PCR analysis of the duplicated region. Individual phenotypes were not available for the Hua-Tung breed and the real-time PCR assay indicated that one bird was homozygous wild-type which is fully possible since *Pea-comb* is not fixed in this breed; furthermore, this bird did not carry the *Pea-comb* haplotype. The results for each individual sample are compiled in Table S3. RJF, red junglefowl; BR, broiler; CGP, Czech Golden Pencilled; FF, Friesian Fowl; FL, Finnish Landrace; RV, Red Villafranquina; TNN, Transylvanian Naked Neck; WL, White Leghorn; FPC, French Pea-comb; HT, Hua-Tung; ORL, Orlov.

*Pea-comb* has previously been assigned to chromosome 1 [9],[10]. We refined the localization by linkage analysis using a dense set of genetic markers and a large segregating family. The interval harbouring *Pea-comb* was defined as 67,831,796–68,456,921 bp on chromosome 1, based on flanking markers showing recombination with *Pea-comb* (Table 1). This interval contains a single gene, *SOX5*, a member of the *SRY*-related HMG box family of transcription factors. *SOX5* is located in a one Mb gene desert that is enriched for Evolutionary Conserved Non-coding Sequences (ECNS; Figure 2A). This is a typical feature of developmentally important genes [11],[12]. *SOX5* was not an obvious candidate gene for *Pea-comb* but the comb is composed of extracellular matrix and *SOX5* has a well-established role in chondrocyte development and production of extracellular matrix [13]. Mouse *SOX5* knockouts die at birth from respiratory distress caused by a cleft secondary palate and narrow thoracic cage [13]. Mouse *SOX5/SOX6* double knockouts die *in utero* with severe skeletal dysplasia, demonstrating that these two genes have critical, redundant roles during development [13],[14].

**Table 1:** Two-point linkage analysis between *Pea-comb* and SNP markers on chicken chromosome 1.

Marker^1^	Position (bp)	Recombination fraction
ARNTL2	70,313,818	0.10
ST8-300	68,456,921	0.02
SOX5.2	68,081,690	0.00
SOX-190	67,891,828	0.00
SOX-220	67,861,996	0.00
SOX-250	67,831,796	0.01
SOX-350	67,731,811	0.01
SOX-800	67,281,780	0.03

1 SNP markers are defined in Table S1.

### Identical-by-Descent (IBD) Mapping Locates the Critical *Pea-comb* Region

To further refine the localization of *Pea-comb* we characterized *SOX5* haplotype patterns among three breeds of chicken, a French experimental population, the Russian Orlov and the Chinese Hua-Tung. These breeds all carry *Pea-comb* and, to the best of our knowledge, there has been no exchange of genetic material between them for 100 generations or more. The Orlov and Hua-Tung are not fixed for *Pea-comb*, allowing recombination to reduce the size of the shared haplotype associated with the mutation. Initial IBD mapping using 12 samples from the three different populations revealed a completely shared haplotype between 67,961,701 bp and 68,061,854 bp (Table 2). SNP genotyping of all Hua-Tung and Orlov individuals available narrowed the shared haplotype further to a 50 kb region spanning positions 67,985,285 bp and 68,035,337 bp (Figure 2A; Table 2). The upstream break-point (67,985,285 bp) was identified using a single Hua-Tung bird. The break was confirmed in two additional individuals from the same population which were homozygous at the six SNPs diagnostic of the *Pea-comb* haplotype, but heterozygous at this break-point. Downstream, the haplotype was broken at 68,035,337 bp in three Orlov birds (Table 2).

**Table 2:** Identical-by-Descent analysis of *Pea-comb* haplotypes from three different breeds in comparison with wild-type haplotypes.

Breed	n**	Markers*																								
		S−190	S−150	S−120	S−100	S−90	S−80	S−74	S−70	S−69	S−65	S−62	S−55	S−50	S−47	S−43	S−30	S−20	S−15	S+10	S+60	S+100	S+130	S+140	S+200	S+260
		C	T	A	*A*	*C*	*T*	*T*	*T*	*T*	*T*	*A*	*A*	*C*	*G*	A	G	A	C	C	C	G	-	-	K	R
**Pea-comb**																										
Hua Tung	2/34	0.50	0.50	#0.00	#0.75	0.77	0.75	0.75	0.97	1.00	1.00	1.00	0.75	0.75	0.77	0.83	1.00	#0.00	#0.00	#0.00	#0.00	1.00	-	-	-	-
Orlov	2/27	0.50	1.00	1.00	0.89	0.88	0.75	0.75	0.90	1.00	0.75	1.00	1.00	1.00	#0.57	#0.61	0.75	#0.00	#0.00	#0.00	#0.00	#0.00	-	-	-	-
French Pedigree homozygotes	4/16	1.00	1.00	1.00	1.00	1.00	1.00	1.00	1.00	1.00	1.00	1.00	1.00	1.00	1.00	1.00	1.00	1.00	1.00	1.00	1.00	1.00	1	1	0.91	0.91
**Wild type**																										
French Pedigree***	2	1.00	1.00	0.25	0.00	0.00	0.00	0.25	0.00	0.50	0.50	-	0.25	0.25	1.00	0.00	1.00	0.00	0.00	0.50	0.00	0.00	-	-	-	-
Friesian fowl	4	-	-	-	-	-	-	-	0.25	-	-	0.38	-	-	0.63	0.25	-	-	-	-	-	-	-	-	-	-
Westfälischer Totleger	4	-	-	-	-	-	-	-	0.50	-	-	0.00	-	-	0.88	0.25	-	-	-	-	-	-	-	-	-	-
Dorking	3	-	-	-	-	-	-	-	0.17	-	-	0.83	-	-	0.17	0.83	-	-	-	-	-	-	-	-	-	-
Houdan	5	-	-	-	-	-	-	-	0.60	-	-	0.50	-	-	0.20	0.50	-	-	-	-	-	-	-	-	-	-
Red Villafranquina	3	-	-	-	-	-	-	-	0.66	-	-	0.33	-	-	0.75	0.00	-	-	-	-	-	-	-	-	-	-
Finnish Landrace	5	-	-	-	-	-	-	-	0.90	-	-	0.30	-	-	0.70	0.20	-	-	-	-	-	-	-	-	-	-
Transylvanian naked neck	3	-	-	-	-	-	-	-	1.00	-	-	1.00	-	-	0.33	0.50	-	-	-	-	-	-	-	-	-	-
Czech Golden Pencilled	5	-	-	-	-	-	-	-	0.30	-	-	0.10	-	-	0.90	0.10	-	-	-	-	-	-	-	-	-	-
Godollo	5	-	-	-	-	-	-	-	0.38	-	-	0.83	-	-	0.50	0.70	-	-	-	-	-	-	-	-	-	-

The observed SNP allele frequencies are given. The alleles defining the minimum shared haplotype among *Pea-comb* birds is indicated in bold italics.

*: The SNP marker names are defined in Table S1.

**: n = the number of individuals used in the analysis. In the case of the Pea-comb birds, the first number indicates the individuals used for IBD mapping by re-sequencing 1 kb fragments, whilst the second number refers to individuals used in the SNP screen with markers underlined.

***: Parental wild-type birds.

# indicates markers where the shared minimum *Pea-comb* haplotype are broken up (i.e. at least one Pea-comb individual homozygous for the allele not associated with *Pea-comb*.

This critical region is located upstream of the first annotated exon however a comparison with *SOX5* from mammalian species indicated that exon 1 is missing from the chicken genome assembly and is expected to be found more than 200 kb upstream of exon 2 (Figure 2A). We confirmed the existence of an upstream exon in chicken by 5′ RACE analysis. The obtained nucleotide sequence (GenBank accession number FJ548639) showed 90% identity to human *SOX5* exon 1, but did not give a match in the chicken genome, implying a gap in the current chicken assembly.

### *SOX5* Mutation Detection Reveals Copy Number Variation

Resequencing the 50 kb region associated with *Pea-comb* from a set of Pea-comb and wild-type birds revealed a limited number of sequence polymorphisms, with fixed differences between genotypes. These potentially causative SNPs were interrogated using a larger set of wild-type birds from the AvianDiv panel [15], however none of the alleles were found to be unique to the *Pea-comb* haplotype (Table 2). The failure to identify a causative point mutation led to a screen of the *Pea-comb* region for structural changes using Southern blot analysis. The SOX-85kb_SB probe (Table S1) revealed a dramatic increase in the hybridization signal of a 3.2 kb *Bam*HI fragment in Pea-comb birds (Figure 2C) whilst other probes from the region gave identical restriction fragment patterns for both alleles. The result implied that *Pea-comb* is associated with a large tandem array of a duplicated sequence containing a *Bam*HI restriction site. PCR and sequence analysis revealed that this DNA fragment is also duplicated on wild-type chromosomes which have two copies (Figure 2B), whereas the *Pea-comb* allele has a large number of copies.

Quantification of the copy number of the duplicated fragment using both pulsed field gel electrophoresis (PFGE) and real-time PCR analysis confirmed that a massive amplification of a duplicated sequence is associated with the *Pea-comb* allele. PFGE analysis using the restriction enzyme *Psh*A1, which cuts outside the duplicated region, gave a 97 kb restriction fragment in Pea-comb birds in contrast to a predicted 10 kb fragment based on the reference genome sequence from a wild-type bird (Figure S1). The result indicates that the *Pea-comb* allele contains about 30 copies of the duplicated sequence. Real-time PCR analysis of Pea-comb birds from three breeds confirmed this finding and revealed a 20- to 40-fold sequence amplification (Figure 2D). The real-time PCR analysis did not indicate two clear groupings corresponding to *Pea-comb* heterozygotes and homozygotes suggesting that the duplication may show further copy number variation among Pea-comb individuals. Interestingly, 100 years ago Bateson and Punnett [16] reported variable expression of the Pea-comb phenotype which may reflect a copy number variation of the duplicated sequence. Although the duplicated sequence is not evolutionary conserved, it is located close to two highly conserved ECNSs (Figure 2A). The distance between these elements is about 10 kb on wild-type chromosomes in contrast to about 100 kb on *Pea-comb* chromosomes. The duplication includes a sequence repeated in two copies on wild-type chromosomes and each copy contains two partial LINE fragments (Figure 2B). The expansion of this duplication must be the causative mutation because it was the only polymorphism showing complete association with the phenotype.

A closer examination of the duplicated sequence shows that it is particularly GC-rich and contains a small CpG island (Figure 2A and 2B). The *wild-type* chromosome contains two copies of this CpG island whereas the *Pea-comb* chromosome contains about 30. This could be relevant for the mechanism of action of this intronic mutation.

### *SOX5* Expression in the Embryonic Nasofacial Region

The Pea-comb phenotype is apparent at hatch and must therefore reflect altered gene expression during development. Tissue samples from the comb region were collected from both homozygous Pea-comb and homozygous wild-type birds at embryonic (E) days 6, 7, 8, 9, 12 and 19 for expression analysis. Quantitative RT-PCR analysis only revealed significant differences in *SOX5* expression at stage E7 and E8 (which were combined due to the low number of E8 samples). The results for E7+8 revealed significant upregulated *SOX5* expression in the comb region in Pea-comb birds (t = −5.0, *p* = 0.002; Figure S2A). Expression analysis was also conducted using primers specific for each exon of *SOX5* (including the previously un-annotated exon 1 described above), however the results did not indicate any difference between genotypes in regards to differential splicing of *SOX5* (Figure S2B).

**Figure 3:**
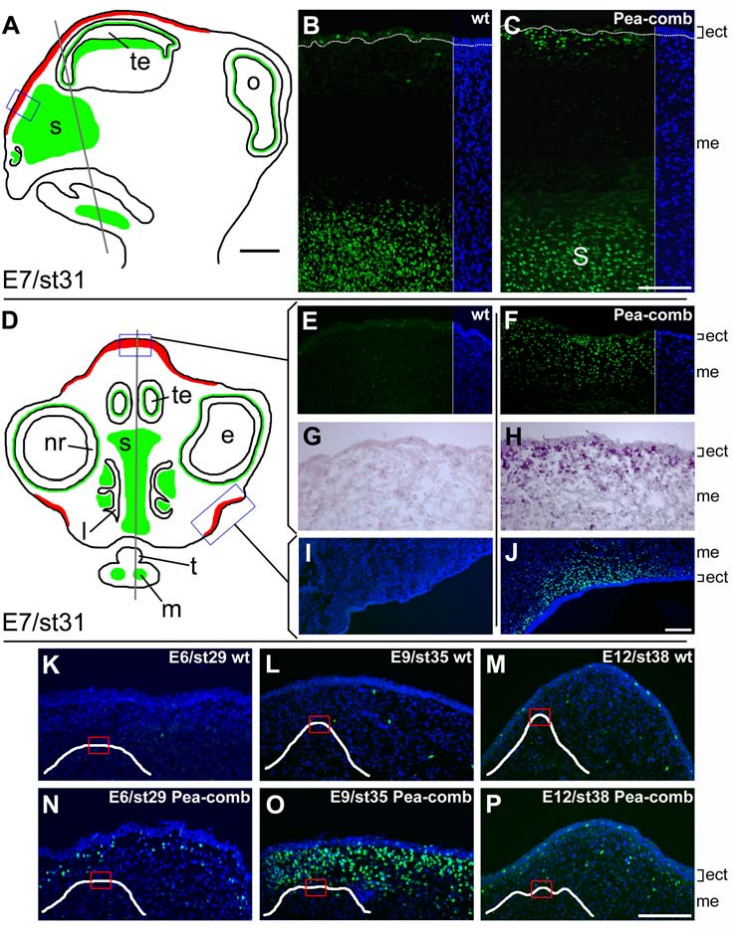
*SOX5* immunostaining in Pea-comb and wild-type embryonic heads. (A, D) Schematic drawings of sagittal and cross-sections of an E7 chick head. Green indicates *SOX5* immunostainings that are identical in wild-type and Pea-comb birds, red indicates *SOX5* staining unique for Pea-comb. The planes of the drawings are shown as shaded lines. Scale bar 1 mm. (B, E, I) Fluorescence micrographs of the wattle and comb regions with *SOX5* immuno- and DAPI nuclear staining of E7 wild-type and (C, F, J) Pea-comb birds. (G, H) Bright-field micrographs of cRNA *in situ* hybridization for *SOX5* mRNA in wild-type and Pea-comb. The positions of the comb and wattle regions shown in panels B, C, E–J are boxed in the schematic drawings. Scale bars 100 μm. (K–P) *SOX5* immuno- and DAPI nuclear staining in the comb region of E6, E9 and E12 wild-type and Pea-comb chickens. Insets show schematic drawings of the comb-ridge shapes in wild-type and Pea-comb. The positions of corresponding fluorescence micrographs are boxed. Scale bar 100 μm. ect; ectoderm, e; eye, l; lumen of nostril, m; Meckel's cartilage, me; mesenchyme, nr; neural retina, o; optic lobe, s; interorbital septum, st; stage according to Hamburger and Hamilton [51], t; tongue, te; telencephalon, wt; wild-type.

Immunohistochemical staining with a human *SOX5* antibody as well as *in situ*-hybridization with a chicken-specific cRNA probe was carried out to investigate *SOX5* expression in both Pea-comb and wild-type embryos during development (Figure 3). Specific immunostaining of nuclei was seen in developing cartilaginous structures including the nasal septum, Meckel's cartilage and optic sclera (Figure 3A and 3D). Scattered and rare SOX5 positive cells were seen in the surface ectoderm (Figure 3B and 3M). All structures with *SOX5* staining in wild-type embryos were also positive in Pea-comb embryos including the scattered cells in the ectoderm. However in Pea-comb embryos, striking ectopic *SOX5* expression was observed in mesenchymal cells located just beneath the surface ectoderm where the comb and wattles will develop (Figure 3A–3J). Differential expression was confirmed with *in situ*-hybridization (Figure 3G–3H) and quantitative real-time PCR (see above). The ectopic expression is transient. Whereas few cells with ectopic expression are visible in the comb region by day E6, they are prominent at E9, and almost completely absent at E12 (Figure 3K–3P). Thus, *Pea-comb* appears to be a spatiotemporal-specific, cis-acting regulatory *SOX5* mutation.

## Discussion

A major challenge in current genome biology is to reveal the biological significance of the many Evolutionary Conserved Non-coding Sequences (ECNS). The analysis of the functional significance of ECNS is hindered by a paucity of mutations in such regions which show an association with a phenotype. Here we demonstrate the first spontaneous *SOX5* mutation associated with a phenotype, despite the rich abundance of ECNS in the *SOX5* region (Figure 2A). *SOX5* is under complex regulation and as demonstrated here, mutations affecting its regulation can have very specific effects. It would be surprising if regulatory mutations in this gene do not to some extent contribute to phenotypic diversity present in humans. For instance, the human face shows a bewildering array of diversity. The nearly identical facial appearances of monozygotic twins imply that this diversity is nearly 100% genetically determined, but knowledge concerning the underlying molecular basis of this diversity is restricted to certain craniofacial abnormalities [17]. It is likely that regulatory mutations in developmentally important genes shape this type phenotypic diversity, and *SOX5* may very well be one of the genes that contributes.

The comb is a sexual ornament that shows strong sexual dimorphism in chickens and the fact that this sexual dimorphism is maintained in Pea-comb birds shows that the Pea-comb tissue maintains the response to the influence of sex hormones (Figure 1). That the comb is under sexual selection is evidenced by red junglefowl females showing mating preferences for males with large combs and reciprocally, males tend to favour females with larger combs [18],[19]. The size of the comb is proportionally larger in many breeds of domestic chickens compared to their wild ancestors. In our previous study of a large intercross between White Leghorn chicken (with larger combs) and red junglefowl, we identified a number of Quantitative Trait Loci (QTL) affecting the size of the comb [20]. Interestingly, one of the QTL controlling the size of the female comb overlaps the *SOX5* locus, which now becomes an obvious candidate gene for this QTL. However, the confidence interval for the QTL is large, as is usually the case in an F_2_ intercross, and the entire *SOX5* region needs to be considered in a search for possible causative mutation(s).

*SOX* genes are defined by their high-mobility-group (HMG) domains and are divided into eight groups (A to H) based on protein sequence comparison [14]. *SOX5* belongs to the D family of *SOX* genes, along with *SOX6* and *SOX13*. SOX5 has been termed an architectural transcription factor [21], as binding to this protein will cause a sharp bend (80–135 degrees) in the bound DNA and may lead to different regulatory regions of a target gene coming into closer proximity. SOX5 has been reported to have a co-operative role in chondrogenesis; during embryonic cartilage formation SOX5 and SOX6 assist SOX9 to activate specific genes [22], and have a repressive role in oligodendrogenesis during neural development [23]. SOX5 is also expressed in the developing neocortex and cranial neural crest during the early stages of development. SOX5 postmitotically regulates migration, axon projection and postmigratory differentiation of certain neocortical neurons [24] but little is known about SOX5 function in neural crest derivatives [25]. With these different roles, the functional consequence of the transient ectopic SOX5 expression in Pea-comb birds is not clear.

The comb is composed of layers of epidermis, dermis and central connective tissue, of which collagen and hyaluronan are the major components [26]. The ectopic SOX5 expression is first seen in E7 (st28) mesenchyme (Figure 3). Previous studies with grafts of comb-primordia from different ages at various locations imply that cells giving rise to the comb are already determined by E4 (st24) [27],[28] and that the determination resides in the mesenchymal components and not in the ectoderm [27]. These experiments also revealed that the morphology of the comb was under control of the mesenchyme [27],[29]. Heterotopic grafts of single-comb primordia to the neck region without beak mesenchyme, lost the serrated single ridge morphology and expanded laterally following the development, resembling that of complex comb types [29] such as the Pea-comb. Hence, changes in the underlying mesenchyme at the time of the ectopic SOX5 expression will not affect the determination and initial stages of the comb development but rather the development of comb shape. Our results indicate that ectopic SOX5 expression changes the modulating properties of the mesenchyme of the nasofacial region beneath the regions of the developing comb and wattles. The serration of a single comb is associated with loosely coherent clusters or points of proliferating mesenchymal cells [30],[31]. Such clusters were not observed in the developing Pea-comb mesenchyme and this difference may be due to the ectopic SOX5 expression.

Pea-comb is an additional example of a Copy Number Variation (CNV) associated with a phenotype. About 12% of the human genome contains tandem duplications that may show CNV [32] and a number of human diseases have been reported to be associated with CNVs [33],[34]. It is important to distinguish CNVs that are due to duplications of single copy sequences (*de novo* duplications) and expansions or contractions of already duplicated sequences. We have previously reported three *de novo* duplications associated with phenotypic traits in domestic animals, Dominant white colour in pigs [35], the Ridge phenotype in Ridgeback dogs [36] and Greying with age in horses [37]. In contrast, *Pea-comb* and most human diseases associated with CNVs involve expansions or contractions of existing duplications. *Pea-comb* is however an unusual CNV associated with a phenotype because it involves the amplification of a non-coding region located far from any coding sequence. *Pea-comb* therefore to some extent resembles the massive amplification of a trinucleotide repeat in intron 1 of *Frataxin* causing Friedrich ataxia [38]. However, the mechanism of action is probably very different since the expansion of the trinucleotide repeat in *Frataxin* leads to the formation DNA triplexes and “sticky DNA” causing transcriptional silencing [38].

The duplicated sequence in intron 1 of *SOX5* is not evolutionary conserved between birds and mammals. This does not exclude the possibility that it contains regulatory elements which are important for SOX5 in birds, or in birds that develop combs and wattles. However, even if the duplicated sequence *per se* is not functionally important, the massive amplification of this sequence may disturb the action of regulatory elements in the region. For instance, tandem repeats may recruit DNA methylation which abolishes protein-DNA interaction at regulatory elements [39]. Our observation that the duplicated region is not only particularly GC-rich, but contains a small CpG island which becomes repeated about 30 times on the *Pea-comb* chromosome, suggests that DNA methylation maybe a plausible mechanism for *Pea-comb* as this effect may spread to neighbouring regulatory sites.

Genetic studies of phenotypic diversity in domestic animals provide a strong case for the evolutionary significance of regulatory mutations. Other examples of cis-acting regulatory mutations underlying phenotypic traits in domestic animals include (i) a nucleotide substitution in intron 3 of *IGF2* with a prominent effect on muscle growth in the pig [40], (ii) regulatory mutations in the gene for microphtalmia-transcription factor (MITF) causing white spotting in dogs [41], (iii) regulatory mutation(s) in *BCDO2* causing the yellow skin phenotype in chicken [6], (iv) a 4.6 kb duplication in intron 6 of *STX17* causing Greying with age in horses [37], (v) an 11.7 kb intergenic deletion causing intersexuality and lack of horns in goats [42] and (vi) a mutation creating an illegitimate microRNA target site in the sheep *myostatin* gene promoting muscle growth [43]. Furthermore, the ridge phenotype in dogs [36] and the dominant white colour in pigs [35] are caused by large duplications that most likely lead to dysregulated expression of some fibroblast growth factor genes and the KIT receptor, respectively. Most of these examples concern growth factors, growth factor receptors, or transcription factors that have important roles during development and for which null mutations are lethal or sub-lethal. The significance of regulatory mutations is also supported by the identification of mutations underlying morphological variation in *Drosophila* [44],[45] and stickleback fish [46]. This wealth of data now demonstrates the prominent role of regulatory mutations, at least for morphological evolution, as predicted by King and Wilson more than 30 years ago based on the limited divergence in protein sequences between human and chimpanzee [47].

## Methods

### Animals

DNA samples from a French pedigree consisting of 7 parental, 14 F_1_ and 244 F_2_ progeny were used for linkage analysis. The parentals consisted of four heterozygous Pea-comb birds and three homozygous wild-type birds. DNA samples from Pea-comb birds for identical-by-descent mapping came from a French experimental population kept by INRA, from a Chinese Hua-Tung population and from the Russian Orlov breed. DNA samples from various domestic breeds collected by the AvianDiv project [15] were used for real-time PCR analysis and to test whether candidate causal mutations from the *Pea-comb* region could be excluded since they were present among birds homozygous for the wild-type allele at the *Pea-comb* locus.

### Linkage analysis

Linkage analysis was conducted using the SNPs compiled in Table S1. SNP genotyping was performed with Pyrosequencing (See ‘Linkage primers’, Table S1 for details). Fine-mapping was carried out on a small number of recombinant individuals that more exactly defined the *Pea-comb* region. In this case, one kb fragments were amplified and sequenced to detect SNPs (see ‘1 kb fragment analysis’, Table S1 for primers).

### Identical-by-Descent (IBD) mapping

IBD mapping was initially performed on a panel of 12 chickens; two Pea-comb and two wild-type birds from the linkage pedigree, four homozygous Pea-comb birds from the French pedigree, two Pea-comb birds from the Chinese Hua-Tung population and two Pea-comb birds from the Russian Orlov population. A collection of one kb regions spanning approximately 67,891,800 bp to 68,181,677 bp on chromosome 1 were sequenced for each animal to identify SNPs between lines (See ‘SNPs used for IBD Mapping’, Table S1, for exact positions). In a similar way, the heterozygosity of chromosome 1, fragment 68,181,600 bp to 68,335,500 bp, was determined by sequencing 16 homozygous Pea-comb birds belonging to the linkage pedigree (Primers SOX+130, SOX+140, SOX+200, SOX+260 in Table S1). This re-sequencing effort revealed potential causative SNP that were differentially segregating between the Pea-comb and non-Pea-comb populations. These polymorphisms were subsequently tested in the non-Pea-comb individuals from the AvianDiv panel and used to define the *Pea-comb* region by six loci, positions 68,038,060 bp, 68,035,337 bp, 68,019,518 bp, 68,011,661 bp, 67,991,941 bp and 67,985,285 bp respectively. Pyrosequencing was used to assay these six variations in 34 Hua-Tung Pea-comb birds and 27 Orlov Pea-comb birds (See ‘Pyro SNPs used for IBD mapping’, Table S1). Lastly, four of these loci were also genotyped for a variety of birds from the AvianDiv panel to check the frequency of the *Pea-comb* haplotype among wild-type chromosomes.

### Real-time PCR analysis

The copy number of the *SOX5* duplication was evaluated by comparing eight populations with wild-type phenotype (red junglefowl, *n* = 5; commercial broiler, *n* = 5; Czech Golden Pencilled, *n* = 5; Friesian Fowl, *n* = 5; Finnish Landrace, *n* = 5; Red Villafranquina, *n* = 5; Transylvanian Naked Neck, *n* = 5; White Leghorn, *n* = 5) to three breeds segregating for *Pea-comb* (French Pea-comb, *n* = 3; Hua-Tung, *n* = 13; Orlov, *n* = 13). The real-time PCR assay contained TaqMan Gene Expression Master Mix (Applied Biosystems), 900 nM of each primer combined with 250 nM of fluorometric probe and 30 ng of genomic DNA. The *SOX5* assay was normalised using an assay designed to ribosomal protein S24 (rps24). Primer and probe concentrations of those reactions were 750 nM and 300 nM, respectively. Each assay was performed in triplicate, averaged and referenced to a wild-type red junglefowl. Details of primer and probe sequences are in Table S2. Fold change was calculated using the equation 2^−(Normalized Ct peacomb assay−Normalized Ct rps24 assay)^ and the range of this value was determined from the combined standard errors of both assays.

### Resequencing

Seventy kb on chromosome 1 from 67,969,741 bp to 68,041,242 bp were re-sequenced using a panel of ten birds; two wild-type parental birds from the linkage pedigree, two red junglefowl (RJF) birds, two homozygous Pea-comb from the French pedigree, two Pea-comb Hua-Tung birds and two Pea-comb Russian Orlov birds. Primers pairs were used to generate over-lapping PCR amplicons ranging from approximately 1200 bp to 1400 bp in size. Internal primers were used with each primer pair set. Primers were designed using Primer3 [48]. DNA sequences were analysed and edited in Codoncode Aligner (CodonCode, Dedham, MA). The RJF genomic sequence used to generate the chicken genome sequence was used as a reference for alignment.

The chicken genome reference sequence contained three gaps. Gap 1 spanned 67,981,199 bp–67,983,790 bp; gap 2, 68,002,231 bp–68,003,557 bp and gap 3, 68,006,200 bp–68,006,994 bp. Gaps 1 and 3 were closed using a PCR-based 2-step strategy [49] (Primers Dynal-75_gap and Dynal-105_gap primers in Table S1), whilst gap 2 was covered using long range PCR (Primers LR_gap1, Table S1). Gap 2 was found to be a tandem duplication, part of the duplication linked to the *Pea-comb* mutation. Therefore sequencing was performed after the amplicon was cleaved with *Xho*I, and both halves sequenced independently.

### Southern blot analysis

Southern blot analysis was performed using a set of six different probes (SOX-55kb_SB to SOX-105kb_SB, Table S1) on a panel consisting of three homozygous Pea-comb birds from the linkage pedigree, three red junglefowls, two commercial broiler samples and two White Leghorn birds. The DNA was digested with *Bam*HI and separated by 0.7% agarose gel electrophoresis.

### Pulsed Field Gel Electrophoresis (PFGE)

DNA plugs were prepared from nine chickens, three of each wild-type, Pea-comb heterozygous and Pea-comb homozygous birds. The plug preparation and restriction digest protocol follows that of Giuffra et al. [35], with the following modifications. Whole blood stored in 0.5 M EDTA was used as starting material and resuspended to a concentration of 25×10^8^ cells/ml in PBS after washing. Plugs were solidified at room temperature prior to digestion for 24 hours at 50°C in 0.5 mg/ml proteinase K, 1×NDS (0.5 M EDTA, 0.01 M Tris, 0.34 M N-Laurylsarcosine, pH 8.0) with constant shaking. Enzyme digestions were performed as described [35]. *Psh*A1 (New England BioLabs) was selected for this experiment as this restriction enzyme was predicted to cut at position 67,998,520 bp and 68,005,614 bp, i.e. outside the duplicated region.

PFGE of the *Psh*A1 digested plugs was performed in a 1.0% agarose gel, 0.5% TBE at 14°C, 6 V/cm, switch times ramped from 1–25 seconds for 17 hours and fragment sizes were estimated using the MidRange I PFG Marker (New England BioLabs). Southern blot analysis was performed as before, using the 986 bp product from the SOX-85kb_SB amplicon (Table S1) as probe.

### Duplication re-sequencing and analysis

The duplicated region was amplified with long-range PCR primers (SOX-Duplication_LR1_F and R, Table S1). In addition, internal primers were used to check the length of the potential duplication through nested PCR of the initial amplicon (Primers SOX-Duplication_F, R11, 12 and 13, Table S1).

### Immunostaining

Heads from staged embryos were fixed in 4% paraformaldehyde in phosphate buffered saline (PBS) for one hour at 4°C. Fixed heads were incubated overnight in 30% sucrose in PBS at 4°C, embedded in OCT freezing medium (Tissue-Tek, Sakura), frozen and sectioned in a cryostat. Cross sections and sagittal sections, 10 μm thick, were collected on glass slides (Super Frost Plus, Menzel-Gläser). The sections were rehydrated in PBS for 15 min and then blocked in PBS containing 1% fetal calf serum, 0.1% Triton-X and 0.02% Thimerosal. The *SOX5* antibody (Abcam, a_6226041) was diluted 1∶500 in blocking solution and incubated on the slides over night at 4°C. The secondary antibodies (Jackson Immunoresearch Laboratories) were incubated at room temperature for two hours at a 1∶200 dilution in blocking solution. Samples were analysed using a Zeiss Axioplan2 microscope equipped with Axiovision software. Images were formatted, resized, enhanced and arranged for publication using Axiovision and Adobe Photoshop.

### *In situ* hybridization

A cRNA probe was made using a DIG RNA labeling kit (Roche). The SOX5 probe was made from the chEST752i6 cDNA clone acquired from the BBSRC ChickEST Database [50]. The probe was hybridized to untreated sections over night at 66°C under conditions containing 50% formamide and 5×SSC in a humidified chamber. The DIG labeled nucleotides were detected using an alkaline-phosphatase coupled anti-DIG antibody (Roche) followed by incubation with BCIP/NBT developing solution (Roche) for 1–5 hours at 37°C. Images were captured using a Zeiss Axioplan2 microscope equipped with Axiovision software (3.0.6.1, Carl Zeiss Vision GmbH).

### qPCR analysis of tissue samples

Tissue was collected from homozygous Pea-comb birds and homozygous wild-type birds. The ages of the birds sampled were embryonic (E) days 6, 7, 8, 9, 12 and 19 (with hatching occurring at approximately day 21). Two Pea-comb and two normal individuals were collected from each stage, with the exceptions of E7, where nine samples (four Pea-comb and five wild-type) were used and two E8 samples (one of each type). Tissues were initially stored in RNALater (Ambion), with total RNA extracted from embryonic tissues using the Trizol reagent (Invitrogen). The most central part of the presumptive beak and comb were dissected out. cDNA was made from 1 μg of RNA using GeneAmp (Applied Biosystems). Samples were run in triplicate using IQ SyBr Green Supermix (Biorad) and normalized to β-actin and TATA-box binding protein (TBP); primers are given in Table S1. *SOX5* was amplified using primers SOX5_cDNA_1 crossing intron/exon boundaries. Control cDNA reactions containing primers but no RNA were performed in parallel. Samples were run on two separate machines: the ABI 7900HT and the Corbett Rotor-Gene 6000. In addition to these primers, primers for each individual exon (2 to 15) were also used to analyse potential alternate *SOX5* splicing in tissue from the comb. These were used on cDNA from two E7 samples (Pea-comb and wild-type) and two E9 samples (Pea-comb and wild-type). Statistical analysis was performed by first correcting Ct values for batch effects caused by using two different machines, then conducting a two-sample t-test on the average of each set of triplicates.

### Web reference

Information on the chicken genome sequence is available at http://www.genome.ucsc.edu.

### Accession numbers

The sequence data presented in this paper have been submitted to GenBank with the following accession numbers FJ548629-FJ548639

## Supporting Information

Figure S1 PFGE Southern blot and real-time PCR analysis of the same chicken samples.
(0.87 MB TIF)

Figure S2 qPCR analysis of SOX5 expression.
(0.30 MB TIF)

Table S1 Primers used for SNP analysis using pyrosequencing, DNA sequencing, 5′RACE experiments and preparation of probes for southern blot analysis.
(0.32 MB DOC)

Table S2 Primers and TaqMan probes for real-time PCR analysis of chicken *SOX5*.
(0.03 MB DOC)

Table S3 Results of real-time PCR analysis of the duplicated fragment in single comb and Pea-comb chickens.
(0.08 MB DOC)
