# Supplementary figures and images for: Copy Number Variation in Intron 1 of SOX5 Causes the Pea-comb Phenotype in Chickens
Source: PLoS Genet. 2009 Jun 12;5(6):e1000512. doi: 10.1371/journal.pgen.1000512 (PMC2685452; doi:10.1371/journal.pgen.1000512)

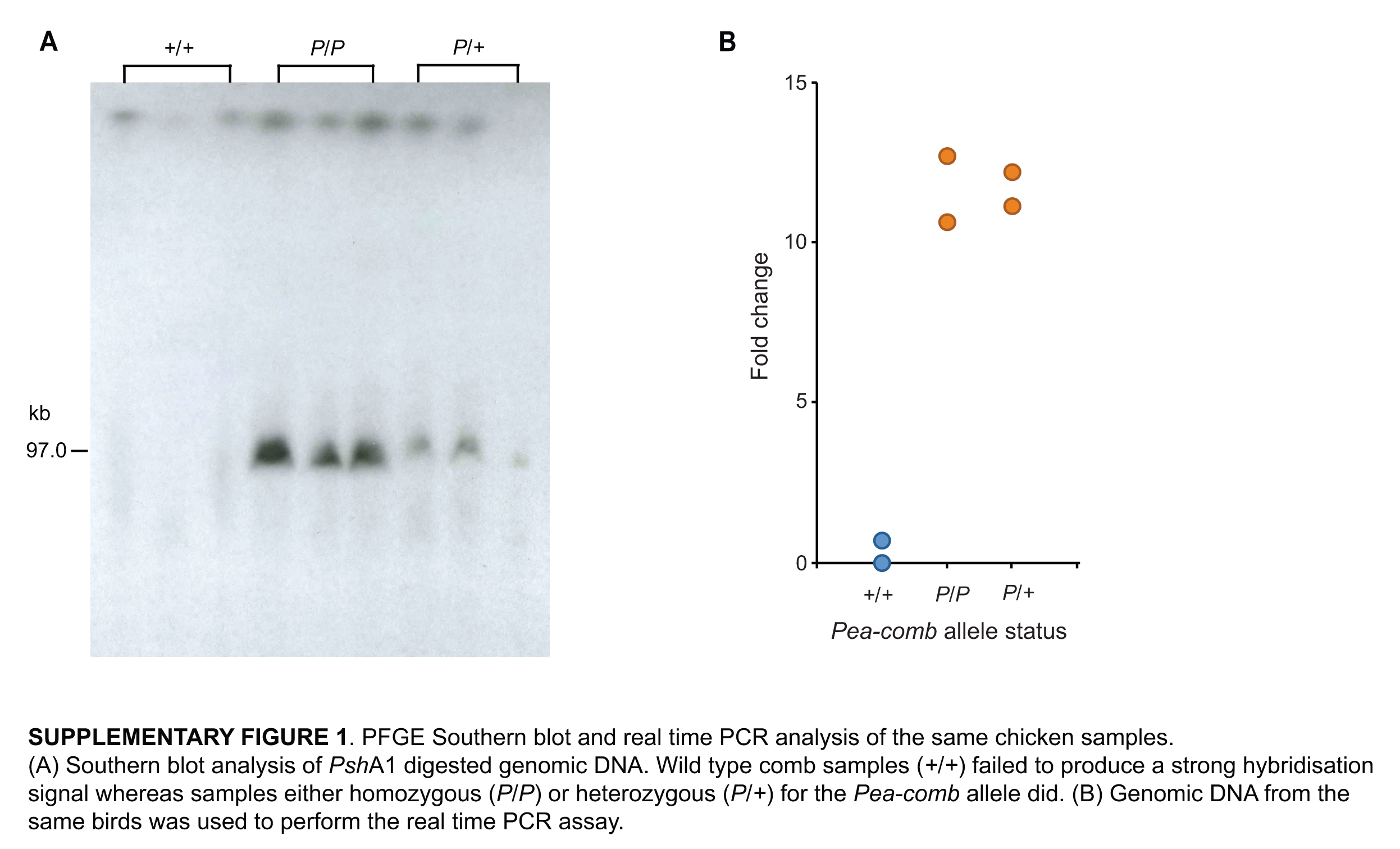

Supplement: Figure S1 — PFGE Southern blot and real-time PCR analysis of the same chicken samples. (0.87 MB TIF) [file pgen.1000512.s001.tif]

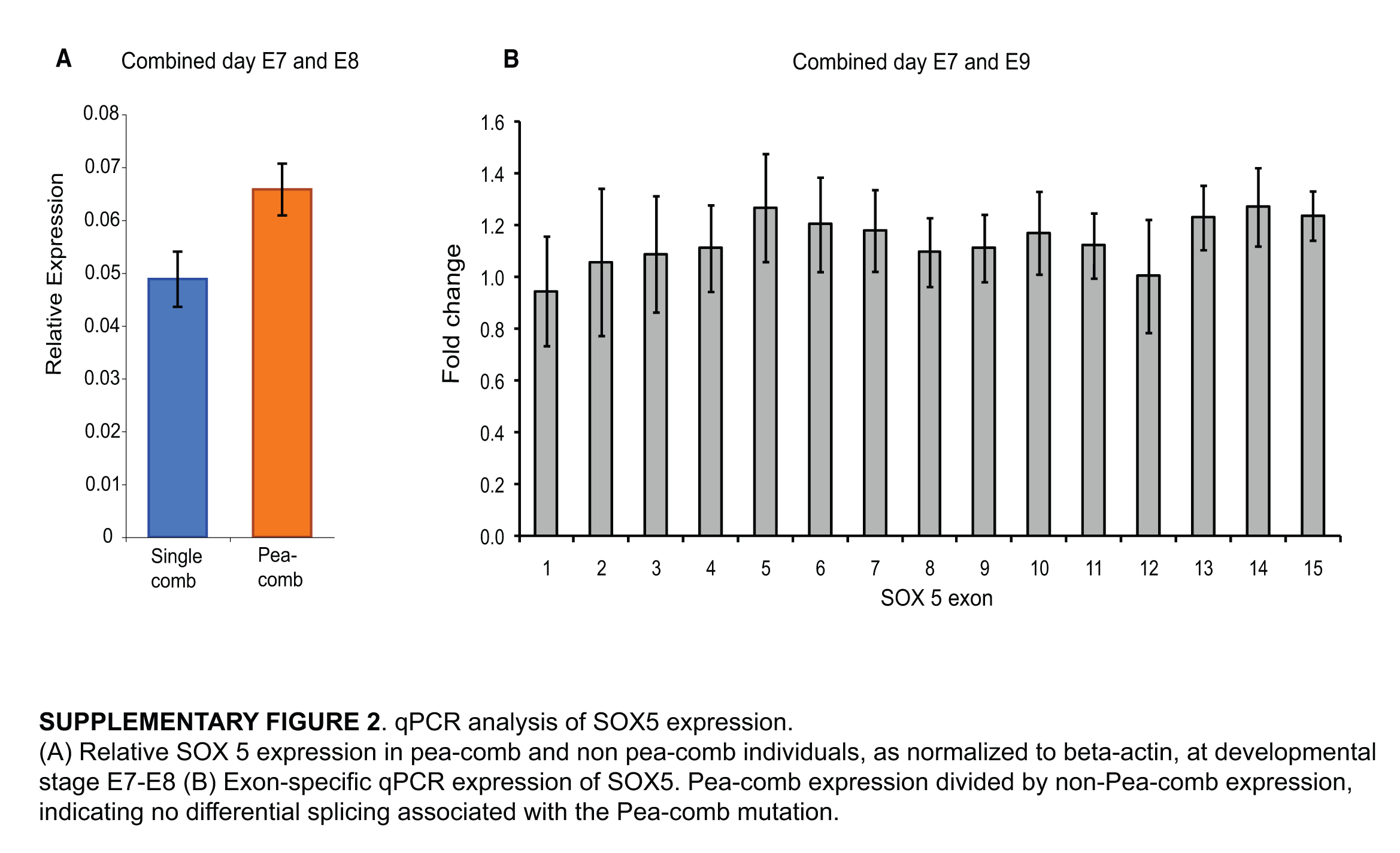

Supplement: Figure S2 — qPCR analysis of SOX5 expression. (0.30 MB TIF) [file pgen.1000512.s002.tif]
