# Supplementary material for: Copy Number Variation in Intron 1 of SOX5 Causes the Pea-comb Phenotype in Chickens
Source: PLoS Genet. 2009 Jun 12;5(6):e1000512. doi: 10.1371/journal.pgen.1000512 (PMC2685452; doi:10.1371/journal.pgen.1000512)
Supplement: Table S1 — Primers used for SNP analysis using pyrosequencing, DNA sequencing, 5′RACE experiments and preparation of probes for southern blot analysis. (0.32 MB DOC) [file pgen.1000512.s003.doc]

Supplementary Table S1. Primers used for SNP analysis using pyrosequencing, DNA sequencing, 5’RACE experiments and preparation of probes for Southern blot analysis

| **Linkage Analysis Primers** | | | | |  | | |  | | |  | | |  | | |
| --- | --- | --- | --- | --- | --- | --- | --- | --- | --- | --- | --- | --- | --- | --- | --- | --- |
| **Pyro Primers** |  | |  |  |  | | |  |  | |  |  | |  | |  |
| **Primer** | **Sequence** | | | | **SNP position** | | | **Ref seq** | | | **Pea(French)** | | | **non-pea(French)** | | |
| SOX-800_Pyro_F | AAAGCAGAACCACTTGCACTTT biotin | | | | 67281818 | | |  | | |  | | |  | | |
| SOX-800_Pyro_R | GAGTGTGCAAGTCCAGTACGTC | | | | 67281818 | | |  | | |  | | |  | | |
| SOX-800_Pyro_seq | GACCTATGCTGGTTATGGTGG | | | | 67281818 | | | C | | | T | | | C | | |
| SOX-400_Pyro_F | CAAAGGCCTTCATAGGACCATA-biotin | | | | 67681679 | | |  | | |  | | |  | | |
| SOX-400_Pyro_R | GCTGATGGTTGGACTGGATAAT | | | | 67681679 | | |  | | |  | | |  | | |
| SOX-400_Pyro_seq | GGTCTTTTCAACCTTTAGTGATTC | | | | 67681679 | | | A | | | G | | | A | | |
| SOX-350_Pyro_F | AATGCATTCTGCATCCATAGC biotin | | | | 67731859 | | |  | | |  | | |  | | |
| SOX-350_Pyro_R | CAAAGCAGTAAATGTGCCTCTG | | | | 67731859 | | |  | | |  | | |  | | |
| SOX-350_Pyro_seq | TGTTATATCCCTTGCAGCTAGAAG | | | | 67731859 | | | G | | | A | | | G | | |
| SOX-5.2_Pyro_F | TTGAAGTGTGTGTGTTCAGCAA | | | | 68081690 | | |  | | |  | | |  | | |
| SOX-5.2_Pyro_R | TATTGCATAAACTTGGCACTGG biot | | | | 68081690 | | |  | | |  | | |  | | |
| SOX-5.2_Pyro_seq | TGTAAGTGAGAGTCATGTTTGGCAT | | | | 68081690 | | | G | | | G | | | A | | |
| ST8_Pyro_F | CCACTTCCCTGTATGTTTTTCC | | | | 68757684 | | |  | | |  | | |  | | |
| ST8_Pyro_R | AGGGCTCCTTTTCTTTAGCACT | | | | 68757684 | | |  | | |  | | |  | | |
| ST8_Pyro_seq | AGATCGTTTTACCACCAGATAC | | | | 68757684 | | | G | | | G | | | C | | |
| ARNTL2_Pyro_F | TCTGTTGCGCTATGCTATGAAT biot | | | | 70313817 | | |  | | |  | | |  | | |
| ARNTL2_Pyro_R | GAGATAAGCGATGTCTTCTTGC | | | | 70313817 | | |  | | |  | | |  | | |
| ARNTL2_Pyro_Seq | CAGATTGAATAAAGTCACAAACACAA | | | | 70313817 | | | C | | | C | | | T | | |
| 1kb Fragment Analysis Primers | | |  |  |  | | |  |  | |  |  | |  | |  |
| **Primer** | **Sequence** | | | | **SNP position** | | | **Ref seq** | | | **Pea(French)** | | | **non-pea(French)** | | |
| SOX-250_F | CCTTGCCTTCCTCAGACTTAGA | | | | 67831900 | | |  | | |  | | |  | | |
| SOX-250_R | GCAAAGAAACAAATCCTCATCC | | | | 67831900 | | | A | | | G | | | G | | |
| SOX-220_F | AGTGTCTGGTGGATGTTTTGTG | | | | 67862467 | | |  | | |  | | |  | | |
| SOX-220_R | GAGAAAACAGGAACATGGAAGG | | | | 67862467 | | | T | | | T | | | C | | |
| SOX-190_F | GATATTTCTCCCCAGAATGCAG | | | | 67891879 | | |  | | |  | | |  | | |
| SOX-190_R | ATCGCCAACATTTTCAAGAACT | | | | 67891879 | | | T | | | C | | | T | | |
| ST8-300_F | TTGCAACATAATCACCACCATT | | | | 68456920 | | |  | | |  | | |  | | |
| ST8-300_R | CTTGCACACTTCAGGAGACTTG | | | | 68456920 | | | C | | | T | | | C | | |
|  |  | |  |  |  | | |  |  | |  |  | |  | |  |
| **SNP primers used for Identical-by-Descent mapping (1kb Fragment primers)** | | | | | | | | | | | | |  | |  | |
| **Primer** | | **Sequence** | | | | **SNP position** | **Ref seq** | | | **Pea(French)** | | | **Pea(HT)** | | **Pea(Or)** | |
| SOX-150_F | | CTGCTGCCCTTTCCTAAACTAA | | | | 67931681 |  | | |  | | |  | |  | |
| SOX-150_R | | GGCTTCCTTACACTGTCCAAAC | | | | 67931681 | T | | | T | | | Y | | T | |
| SOX-120_F | | TGATCTGTGTTCACCCTAGCAC | | | | 67961784 |  | | |  | | |  | |  | |
| SOX-120_R | | AAATGAAAAAGGGAAGGGGTAA | | | | 67961784 | G | | | A | | | G | | A | |
| SOX-80_F | | GAGAAGTGGGTGTAGCCAGACT | | | | 68004654 |  | | |  | | |  | |  | |
| SOX-80_R | | GCTACACTGTTTGCCCCTAATC | | | | 68004654 | T | | | T | | | T | | T | |
| SOX-74_F | | TCTGTTTTGTTTAGGGGGAGAA | | | | 68008573 |  | | |  | | |  | |  | |
| SOX-74_R | | GCAATGCAGAATAGGGGTTTAG | | | | 68008573 | C | | | T | | | T | | T | |
| SOX-69_F | | ACGTTTACCAGCCACTCAATTT | | | | 68012275 |  | | |  | | |  | |  | |
| SOX-69_R | | ACCTGAAGGAAACTGAACCAGA | | | | 68012275 | T | | | T | | | T | | T | |
| SOX-65_F | | TCTGAGTCTTTCTTCCCTCCAG | | | | 68016723 |  | | |  | | |  | |  | |
| SOX-65_R | | CTCTTGCCAACAAATGCTACAG | | | | 68016723 | C | | | T | | | T | | T | |
| SOX-55_F | | GCTTCAGGGAGTGCTACATTCT | | | | 68026570 |  | | |  | | |  | |  | |
| SOX-55_R | | GAAAAGCAAAAAGCAGAGGAAA | | | | 68026570 | G | | | A | | | A | | A | |
| SOX-50_F | | GCATTTCCTCACTGTGTGATGT | | | | 68032016 |  | | |  | | |  | |  | |
| SOX-50_R | | TCGTGATCCAAAATACATCTGC | | | | 68032016 | C | | | C | | | C | | C | |
| SOX-30_F | | GAAGGATGTTCTTACCCGTTTG | | | | 68052081 |  | | |  | | |  | |  | |
| SOX-30_R | | TCATAAGCCATGCTAGGGATCT | | | | 68052081 | G | | | G | | | G | | G | |
| SOX-20_F | | AGTCACTTACTGGGGTGAGGAA | | | | 68061735 |  | | |  | | |  | |  | |
| SOX-20_R | | ATGAAGCAAGGCCAAAAGAATA | | | | 68061735 | A | | | A | | | G | | G | |
| SOX-15_F | | CGTCATGGTGTACAGACCACTT | | | | 68067025 |  | | |  | | |  | |  | |
| SOX-15_R | | TAATGATGGAGAGCCAAAACCT | | | | 68067025 | T | | | C | | | T | | T | |
| SOX+10_F | | GGTTGCAACTGGAGCTTAGAGT | | | | 68091982 |  | | |  | | |  | |  | |
| SOX+10_R | | AACCCCAATCTCAAATGTATGG | | | | 68091982 | C | | | C | | | G | | G | |
| SOX+60_F | | GAGTTTGGGTCTCAGCTTGTCT | | | | 68140804 |  | | |  | | |  | |  | |
| SOX+60_R | | GAGGCTGAGCTGGTTTTAATGT | | | | 68140804 | C | | | C | | | T | | T | |
| SOX+100_F | | GTCCCTGTGATCTCGTTCTCTT | | | | 68181532 |  | | |  | | |  | |  | |
| SOX+100_R | | CCCCCAGATTTAATGAAAAACA | | | | 68181532 | A | | | G | | | G | | A | |
| SOX+130_F | | TCAGATTTGTGTTCGTCATTCC | | | | 68210905 |  | | |  | | |  | |  | |
| SOX+130_R | | TTCCCAGTATGCAGCAATACAC | | | | 68211855 | - | | | No Hets | | | - | | - | |
| SOX+140_F | | GCAGGAGGTGAGATAAGAAGGA | | | | 68220448 |  | | |  | | |  | |  | |
| SOX+140_R | | ATCCGACATAAAAGCACTCGAT | | | | 68221419 | - | | | No Hets | | | - | | - | |
| SOX+200_F | | TTCAAAGGTAGCCAGATCCTGT | | | | 68282559 |  | | |  | | |  | |  | |
| SOX+200_R | | ACGGTTGTGCTTAATGTTACCC | | | | 68282559 | G | | | K | | | - | | - | |
| SOX+260_F | | TAACCGAAAACACAGCTAGCAA | | | | 68335331 |  | | |  | | |  | |  | |
| SOX+260_R | | CTCCAGATTCAACAACTGATGC | | | | 68335331 | A | | | R | | | - | | - | |
| **Pyro SNP primers used for Identical-by-Descent mapping** | | | | | |  |  | | |  | | |  | |  | |
| **Primer** | | **Sequence** | | | | **SNP position** | **Ref seq** | | | **Pea(French)** | | | **Pea(HT)** | | **Pea(Or)** | |
| SOX-100_1 Pyro_F | | AGAACAGAGAGAAGGTGGGAAG | | | | 67985285 |  | | |  | | |  | |  | |
| SOX-100_1 Pyro_R | | M13 TGTTGCTTATTCGTCCCTAGAA | | | | 67985285 |  | | |  | | |  | |  | |
| SOX-100_1 Pyro_seq | | GGGCTGTGCAGGATTCCCCACCC | | | | 67985285 | G | | | A | | | A | | A | |
| Pyro SOX-90_F | | CGTTCTGCTTCTGAATTGTTTT | | | | 67991941 |  | | |  | | |  | |  | |
| Pyro SOX-90_R | | M13 GTGTTTGCCTGCACTACTTGT | | | | 67991941 |  | | |  | | |  | |  | |
| Pyro SOX-90_Seq | | AACACACATAATTAGACAAAGTGA | | | | 67991941 | T | | | C | | | C | | C | |
| Pyro SOX-70_F | | M13 CACAGGAAAACCCATTTGACT | | | | 68011626 |  | | |  | | |  | |  | |
| Pyro SOX-70_R+Seq | | GCAACTTTGAGAGAAGCTCAGAA | | | | 68011626 | A | | | T | | | T | | T | |
| SOX-62 Pyro SNP_F | | ATATGATCCCAATTCCAACTGC | | | | 68019518 |  | | |  | | |  | |  | |
| SOX-62 Pyro SNP_R | | M13 ATATGATCCCAATTCCAACTGC | | | | 68019518 |  | | |  | | |  | |  | |
| SOX-62 Pyro SNP_Seq | | ATAAAGTTTTGAAAATGTATTATTAG | | | | 68019518 | C | | | A | | | A | | A | |
| SOX-47 Pyro SNP_F | | CACTCTTTCAGTGGTCTTGCAG | | | | 68035351 |  | | |  | | |  | |  | |
| SOX-47 Pyro SNP_R_M13 | | M13 CCCACAGTATTCCATCAGCTCT | | | | 68035351 |  | | |  | | |  | |  | |
| SOX-47 Pyro SNP_Seq | | ACCTCCAACAGGCAAGATTCTGAAA | | | | 68035351 | G | | | G | | | S | | S | |
| SOX-43 Pyro SNP_Seq | | CTTTGAGTCATGCTCAAC | | | | 68038060 |  | | |  | | |  | |  | |
| SOX-43 Pyro SNP_F | | TTGAGGGAGACTCAGAAAGACC | | | | 68038060 |  | | |  | | |  | |  | |
| SOX-43 Pyro SNP_R | | ACGTACCCTAATGGCACAACTT | | | | 68038060 | G | | | A | | | R | | R | |

| **Sequencing primers** |  |
| --- | --- |
| Primer | Sequence |
| SOX-45-35_F_1 | TGACTACACTTCAGTGCCACCT |
| SOX-45-35_F_2 | GCAAAGTGAAAAGAAAACGGTA |
| SOX-45-35_F_3 | CTGTAAAGACGGTTCCTATCCA |
| SOX-45-35_F_4 | AGTTGAAGGCTTTCTTTTCTGC |
| SOX-45-35_F_5 | CAACGATCTCTGCTTCCTTTCT |
| SOX-45-35_F_6 | TATTATTAGCCAGGGGCTACCG |
| SOX-45-35_F_7 | GAAATTCTTCCGAGCACAAATG |
| SOX-45-35_F_8 | GGCTGGAACTTTTTCTTTCCTC |
| SOX-45-35_R_1 | CTAAAGGCGTTCCTTCATTTTG |
| SOX-45-35_R_2 | CCTTAAGGGCCACTAAAAATGA |
| SOX-45-35_R_3 | ATTTCTCTTGCTCCGTTCCTTT |
| SOX-45-35_R_4 | CATCCCTCTGCTCATACTTGTG |
| SOX-45-35_R_5 | GTCCATGCACACGTTGTAAGC |
| SOX-45-35_R_6 | CGGAAGAATTTCCACTCCTTTA |
| SOX-45-35_R_7 | TTTCCGGAGGAAAGAAAAAGTT |
| SOX-45-35_R_8 | GTTGAGGCGTCCAAAAGTTTC |
| SOX-45-35_IF_1 | TCGGAGCTGCTTTAAAATGAAT |
| SOX-45-35_IF_2 | TTGAGGGAGACTCAGAAAGACC |
| SOX-45-35_IF_3 | CTCCTGAAGGGTAGCGTCAGT |
| SOX-45-35_IF_4 | AAAGGAACGGAGCAAGAGAAAT |
| SOX-45-35_IF_5 | CACAAGTATGAGCAGAGGGATG |
| SOX-45-35_IF_6 | GGGGGAGATTAAATAGGGTAGC |
| SOX-45-35_IF_7 | GCTCTCTCTCTTTTCCCGTATTT |
| SOX-45-35_IF_8 | AATTGGTGATCCCAACCAATAC |
| SOX-45-35_IR_1 | GCATAAGAAGACCCACGAAGAA |
| SOX-45-35_IR_2 | TGCAGCTAACTGGATTTCAAAC |
| SOX-45-35_IR_3 | GCAGAAAAGAAAGCCTTCAACT |
| SOX-45-35_IR_4 | CGTTTAAGCATCTTCAGCACAA |
| SOX-45-35_IR_5 | TTTTCCACAGAGGTTCATTCAT |
| SOX-45-35_IR_8 | GCTAACAACCTGCGACAAACC |
| S-60-45_Pr1_F | TCCACTAAAATAAGGGTGCTCTG |
| S-60-45_Pr2_F | CAAGAGCTAGTTTTGCCTTGCT |
| S-60-45_Pr3_F | AAGTTGTTGAAGCTGTGATTGG |
| S-60-45_Pr4_F | TCAACTTCATTGCTGCTCAGTT |
| S-60-45_Pr5_F | AAACTTCCTTTGTTTGGTGCAT |
| S-60-45_Pr6_F | AGGCTAGCAGTGATTCAAGGAA |
| S-60-45_Pr7_F | GATTGTGCCTAAACTTCCCAAT |
| S-60-45_Pr8_F | TGCTATGGCAGTAGAGGTTGAA |
| S-60-45_Pr9_F | GGCTCTGAGCAGGTGTAGTAGG |
| S-60-45_Pr10_F | CTGGTTTCCTGTTTCCAGACTC |
| S-60-45_Pr11_F | TGAACAACCATTTTGGTACACAG |
| S-60-45_Pr12_F | CCATCATAGAAGACTTAAGAGGGAAA |
| S-60-45_Pr13_F | CTTGGTTGTTGAAAATGGTATAAGG |
| S-60-45_Pr14_F | AGCTTGGGGTGCTTAGTAGCTG |
| S-60-45_Pr1_R | TCAGAAGTGCCTGTATCTCTGC |
| S-60-45_Pr2_R | ACCTAGGAATGTCGGATGCTT |
| S-60-45_Pr3_R | GCAAGCAACTCTGCACATTTTA |
| S-60-45_Pr4_R | TTTGCAGCTCAATGTGAGATTT |
| S-60-45_Pr5_R | GCCAGTGTTGGTCTAGCTTCTT |
| S-60-45_Pr6_R | AAATCTCTTTCTGCAGCCAAAT |
| S-60-45_Pr7_R | CTGCATACCACACACATGAAAA |
| S-60-45_Pr8_R | CAAGCGGTTTTTCTTTCTAAGC |
| S-60-45_Pr9_R | CCTCTAGTCACAAGTAACCACCA |
| S-60-45_Pr10_R | ATTTTCACAAGTGGGTTTCAGC |
| S-60-45_Pr11_R | AATTCTCGGCATCAATTCTACC |
| S-60-45_Pr12_R | GAAATCTCGACCAAACAGGAAG |
| S-60-45_Pr13_R | TTCTCTTGCCATCTAACACTGC |
| S-60-45_Pr14_R | CGAATGCCATTTAGTTCTGTTG |
| S-60-45_Internal_Pr1_F | TGCTGAGTCCAGAATCAAAATG |
| S-60-45_Internal_Pr2_F | GGGTTTATTAATGCTGCAGAGC |
| S-60-45_Internal_Pr3_F | TCTGAAACGTGGCATAGTTGG |
| S-60-45_Internal_Pr4_F | TATTTTTGTCCTTTGCCTTTGC |
| S-60-45_Internal_Pr5_F | TTTCCCATCATTGTTTACATGC |
| S-60-45_Internal_Pr6_F | CCTAGTGTGATTTGACAGTAACACTTC |
| S-60-45_Internal_Pr7_F | TTACTCTTCATGGCTGAACTGC |
| S-60-45_Internal_Pr8_F | TTTTGTTTGATTTAGGCCTTGG |
| S-60-45_Internal_Pr9_F | AATGGATTTTCTGTCTGGAACC |
| S-60-45_Internal_Pr10_F | GCAATAGGCTGTGGCTTTTAGT |
| S-60-45_Internal_Pr11_F | AAAGCTCATCAACCCCTGAAG |
| S-60-45_Internal_Pr12_F | TTGGTGTAGGAAAACACTGCAA |
| S-60-45_Internal_Pr13_F | ATCTCTGCATTATGAGCCCAAG |
| S-60-45_Internal_Pr14_F | TATTTAGGTTGCTTGCGAGGTT |
| S-60-45_Internal_Pr1_R | CAAACAGTCAGGGCATCAGTTA |
| S-60-45_Internal_Pr2_R | CAATTTCTCCTGTTCACCTTCC |
| S-60-45_Internal_Pr3_R | GGGGACTGTATTGCACAATCTT |
| S-60-45_Internal_Pr4_R | GTTTAAGAATGCTGGCAGGAAC |
| S-60-45_Internal_Pr5_R | AACCTCATCTGCTGTCTTCTCA |
| S-60-45_Internal_Pr6_R | TCATTCAAAAGTTCAAGGAGTGG |
| S-60-45_Internal_Pr7_R | AAGAAGAGAGGGCTGCAAAAAT |
| S-60-45_Internal_Pr8_R | AGTACAACCCACCCATAACACA |
| S-60-45_Internal_Pr9_R | AAGTATTTCTTTGGGGCAGTGA |
| S-60-45_Internal_Pr10_R | GCTGAAGAGTCAGATGTTGCTG |
| S-60-45_Internal_Pr11_R | ATTCCAATAGCATGCTCCCTTT |
| S-60-45_Internal_Pr12_R | TACAGTGGTCACGAGCATTTTC |
| S-60-45_Internal_Pr13_R | GAAATCTCGACCAAACAGGAAG |
| S-60-45_Internal_Pr14_R | TTCTCTTGCCATCTAACACTGC |
| SOX-60-45_6k_gap_F | GCCACTCCTTGAACTTTTGAAT |
| SOX-60-45_6k_gap_R | CCTCTGAAGAGAAGAGCTGCTA |
| SOX-60-45_12k_gap_F | GGAAGAAAAAGCAAAAAGACTGC |
| SOX-60-45_12k_gap_R | TGTATGGCTAACCCTGAAATCC |
| SOX-60-45_14k_gap_F | TGTCTTGCTTCCAAACTAAGTGG |
| SOX-60-45_14k_gap_R | GTGTAAGTGTGGGAACACTGGA |
| SOX-65kb-60kb_1_F | GTTTTGGGTGGCTTCACTGT |
| SOX-65kb-60kb_2_F | CCCTGGTTTTATGATGGATTTC |
| SOX-65kb-60kb_3_F | TGCTGTCAGTGCCTTTCTACTC |
| SOX-65kb-60kb_4_F | TGCCAGTGATTAAACAATTACTCTG |
| SOX-65kb-60kb_5_F | CAGAGCTTAACTCCTGGCATTT |
| SOX-65kb-60kb_1_R | CATGACTTCTGCTGGAAATTGA |
| SOX-65kb-60kb_2_R | GGTTTCCTACCCATTTCATGTC |
| SOX-65kb-60kb_3_R | CCACCTTCAAACACCCTTAAAA |
| SOX-65kb-60kb_4_R | GGAGTTAGAAGCATTTAGAAGTAACC |
| SOX-65kb-60kb_5_R | TCTTAGCAAGCTTAAAGAAAGTCTG |
| SOX-65kb-60kb_1_IF | CAGTATTTACTTTCTCTGCAGTTTCG |
| SOX-65kb-60kb_2_IF | ACAGGAAAGGGTCAAATTGTGT |
| SOX-65kb-60kb_3_IF | CGTAAGTAGGCTTACCCTGTGC |
| SOX-65kb-60kb_4_IF | TGGTGAGGTTCATTATGTGGAA |
| SOX-65kb-60kb_5_IF | CAAGCTTAATGGTTGGTGTTGA |
| SOX-65kb-60kb_1_IR | CCCAGAAAGTACCAGCTTAGCA |
| SOX-65kb-60kb_2_IR | AATAAGGCGTTACTGCTCAAGG |
| SOX-65kb-60kb_3_IR | AAACGCAGCAAATTAAGAGCAT |
| SOX-65kb-60kb_4_IR | CCTCCTGAAGAGGCAGTGCT |
| SOX-65kb-60kb_5_IR | TTCCCTGGTAAAGTACAATCTTCC |
| SOX-66-65_1F | TGGTCTCTATGACCAGTGTTTCA |
| SOX-66-65_1IF | CCTTGCAATGCTGTGTGTCA |
| SOX-66-65_1IR | CTCCTTCGACAAAGCGAGATAC |
| SOX-66-65_1R | GGCAGCAGAAAAGAATCAAAAG |
| SOX-66-65_2F | TGACTGGATTTGTCCAGGTAGA |
| SOX-66-65_2IF | ACAGGGCGGGTTTTTAGTATTC |
| SOX-66-65_2IR | TCTTGCTCCACTGTCAGACTTC |
| SOX-66-65_2R | AAGAGGAAGAAGAATGGACGTG |
| SOX-69-66_1F | TGCCCTATAGTGTAGCTTGAATG |
| SOX-69-66_1IF | TAAACTGCTCCAGATGATGCAC |
| SOX-69-66_2F | TGCTGAAAATGTTTACGAGCTT |
| SOX-69-66_1R | CATCTTTATCTCCTTGCAAGACC |
| SOX-69-66_2IR | CATCAGGAATAACCAGGGAGTC |
| SOX-69-66_2R | ACACACATATGCAAAGTGAAAACAC |
| SOX-74-70_1F | GCATAATGCTTTCTCCAGGTGT |
| SOX-74-70_1IF | GTTTCTATGAATGGCTTGTTGG |
| SOX-74-70_1IR | CTTGTTATTATGACAGTGGCACAGAAT |
| SOX-74-70_1R | GCAACTCCCCTCTGAGTCTTAT |
| SOX-74-70_2F | AAATGTGCTGCCACCTATCAC |
| SOX-74-70_2IF | CCTGCATTTCCAATCCATTATT |
| SOX-74-70_2IR | TAACACGCAAAAGGAGTCCTCA |
| SOX-74-70_2R | GTGCATTATCACAACAGCCAAT |
| SOX-75-70_F1 | ACAAGGAAGGCTGTTGGCTA |
| SOX-75-70_IF1 | TTTCCAGCTTTGCTTTAGAAGG |
| SOX-75-70_F2 | TGTTTTATAACGGCCTTTGGAC |
| SOX-75-70_IF2 | TTTGGTAGCGTTATGGATGATG |
| SOX-75-70_F3 | TTGTCCTGGGCTATCCTAAAAG |
| SOX-75-70_IF3 | TGCTTCCCAGAAAGGTCTTCTA |
| SOX-75-70_F4 | ATAGCTTCGCTGGGACTAACTG |
| SOX-75-70_IF4 | GAAGCTGACAGATACACCCTCA |
| SOX-75-70_F5 | ATGTGGGCACACTGTAAGACTG |
| SOX-75-70_IR1 | TCATTAAGTGCTTTTCGGGAGA |
| SOX-75-70_R1 | GCTGCATTGTCTGTCCTGAG |
| SOX-75-70_IR2 | CAGGCCTGTATGATTGACTGAA |
| SOX-75-70_R2 | TAACAGTAGCACGACATGAGAGG |
| SOX-75-70_IR3 | CCTTCCATCTTCTCATCTGCTC |
| SOX-75-70_R3 | CCACATGATAGGGCTTGTCTTA |
| SOX-75-70_IR4 | GCCTTTTAAAGTGCTTCCAAGA |
| SOX-75-70_R4 | GACCATCAATCCTTTCATTTCC |
| SOX-75-70_R5 | AAACACCACCGAATGTTCTTCT |
| SOX-80-75_1F | ACATTTGAAAAGTCGTGGCAGT |
| SOX-80-75_1IF | GGATGGTCACATCCAAACATCT |
| SOX-80-75_1IR | CAATTCATTTCTTACAGCCAACC |
| SOX-80-75_1R | TCTTGGGTCCAGGAGTATGTCT |
| SOX-90-80_1F | CCAAAAGGTTCTGAAGAGTGCT |
| SOX-90-80_1IF | GAAGAGTGCAAGTGTCTGCAATA |
| SOX-90-80_2F | CTGTGCTCAGTATTTAAGACTTTGA |
| SOX-90-80_2IF | AAATGAGGGCTGCAATTAACTC |
| SOX-90-80_3F | TTTGGCAGATAGCGTCCTTATT |
| SOX-90-80_3IF | GCAATTCGCATAACAATTTCCT |
| SOX-90-80_4F | CTGTTCACAAGTGCTTCAAAGG |
| SOX-90-80_4IF | AGGGGAGAAAGGAGTACTGGAT |
| SOX-90-80_5F | CCTTCCACGTGAAGACTGAATG |
| SOX-90-80_5IF | GTTCACAGTAGCGTCCACAAGG |
| SOX-90-80_6F | ACGCAGCAACCTTAACAACTG |
| SOX-90-80_6IF | CCTCAGTGTCACCCCTACATTT |
| SOX-90-80_7F | AGCACTGCATTCGTGTGTCT |
| SOX-90-80_7IF | GAGATATTCAGAAGCTGCCACA |
| SOX-90-80_1IR | TTACCTGTGTTAGGTGCTGGAA |
| SOX-90-80_1R | GGACATGTTTGTGGACTTCAGA |
| SOX-90-80_2IR | ATTACTGCCCCGAAGACCTATT |
| SOX-90-80_2R | ATTTTTGGCTGCCTCTTTTCTC |
| SOX-90-80_3IR | TTAAGCCATTAGAGCTCCGAGT |
| SOX-90-80_3R | GCAAACAGCTTTTGAAACATGA |
| SOX-90-80_4IR | AGCCATACTCCAAGAAATGCTG |
| SOX-90-80_4R | ATGTGAAACACGCATCAGTACC |
| SOX-90-80_5IR | GAGATCCTCGACCCTGTGCT |
| SOX-90-80_5R | GTTCATCTTCATTCCCCATCAG |
| SOX-90-80_6IR | CCCTACAGCCATCACCTGTAA |
| SOX-90-80_6R | CTGGCTACACCCACTTCTCTCT |
| SOX-90-80_7IR | AGACAGGACAACAGCCAAAGTT |
| SOX-90-80_7R | GCAGGGATTGCACTTAAATGAT |
| SOX-90-80_8F | CCCGTCTAATGATGGTGGTAAT |
| SOX-90-80_8R | AGCAGCACAGGAAATGTCAAC |
| SOX-90-66kb_gap1300-13800_F | TCCCCTCTTGGACATAAGGTAG |
| SOX-90-66kb_gap1300-13800_R | AGTAATCGGTGGGCTCTTTTC |
| SOX-90-66kb_gap1907-19188_F | GGCACCAGAGAGAAATGTAAGG |
| SOX-90-66kb_gap1907-19188_R | CCACGAATAACAAAACAAGCAG |
| SOX-90-66kb_gap11645-12199_F | CCTTTCAATGCATAAAGGTGGT |
| SOX-90-66kb_gap11645-12199_R | TTCTTGGGTCCAGGAGTATGTC |
| SOX-90-66kb_gap13036-3309_F | CTCGGAGCTCTAATGGCTTAAA |
| SOX-90-66kb_gap13036-3309_R | TTAGCATTATCACCGTTTGCTG |
| SOX-90-66kb_gap14200-4265_F | TTTTTCCTTCCACGTGAAGACT |
| SOX-90-66kb_gap14200-4265_R | CAATGTAGGCAACAATTCTGGA |
| SOX-100-90_F1 | CGGAAGGGAGCCTTAAAGTATT |
| SOX-100-90_IF1 | AAATCCTGGCTGCTTTTTGAT |
| SOX-100-90_F2 | CTCCTCTCTTCATCCCAGTGAC |
| SOX-100-90_IF2 | AGCAGATGTTGCGTAAACAGAA |
| SOX-100-90_F3 | CCACAGACTGCTGTTTTGTCTC |
| SOX-100-90_IF3 | CATGTTGGAATTGGTTGGATAA |
| SOX-100-90_F3 | TGCAGATGCAAAACTAATCAGC |
| SOX-100-90_IF3 | TCGTTCCAGCGTGATACATAAG |
| SOX-100-90_F4 | GCTACAAAATAAGAGGCATTCTTGA |
| SOX-100-90_IF4 | CCAGACTATCCATCTTCTCCTCA |
| SOX-100-90_F5 | ACTGTTATCCAAAGCGGAAAAA |
| SOX-100-90_IF5 | TGTCATCCTGAGGGATTTACAA |
| SOX-100-90_F6 | TGTAGGTTTCAAAGGAACTGTGAG |
| SOX-100-90_IF6 | AGACTGGATATATTTCTTGTGTGGT |
| SOX-100-90_F7 | GAGAGCACACCAAATTGTCTGA |
| SOX-100-90_IF7 | CTCAGCAGCAAGAACTCTGAAA |
| SOX-100-90_IR1 | GGAGCTCTTCTTCTAGGGACAA |
| SOX-100-90_R1 | AGTTTCGAGGAGGAAAGGAAAC |
| SOX-100-90_IR2 | TCTCTTGCAACACGATAACACC |
| SOX-100-90_R2 | CGAGAGTAATGCCTCCTGTTTT |
| SOX-100-90_IR3 | AGCGTGTCGTATTTCTGAAAGC |
| SOX-100-90_R3 | GCATCATAAAAAGGATGGCAGT |
| SOX-100-90_IR3 | ATCCCCTACCAACACTTTCCTT |
| SOX-100-90_R3 | TGCTTTACTCCAACTCACTGGA |
| SOX-100-90_IR4 | CTGCCATAGCCAGTGCTGAGT |
| SOX-100-90_R4 | GATAATGCACCGACATGCTG |
| SOX-100-90_IR5 | TTTCACAAGGTGATCAGAACTCTC |
| SOX-100-90_R5 | ACTTTCCTGACAGCTCAGATGC |
| SOX-100-90_IR6 | ACTCACTGACCCTTCATCTCTTTT |
| SOX-100-90_R6 | TATGCTTAGAGCCCAGCAGACT |
| SOX-100-90_IR7 | AAGACCTTCAACATCAGTGTGC |
| SOX-100-90_R7 | AGCACTCTTCAGAACCTTTTGG |
| SOX-115-100_F1 | GCCTGAACTGAGTACCCTCCT |
| SOX-115-100_IF1 | AAAGAATCCTCGTCTCCTGTTG |
| SOX-115-100_F2 | CTGGAAATGCCTGACTTTAACC |
| SOX-115-100_IF2 | TTCCTCCATTCCAATTTTTGAC |
| SOX-115-100_F3 | GTTACAGCTTAAAAGGCCCAGA |
| SOX-115-100_IF3 | CTCAGCAGAGCAGTGATGAAA |
| SOX-115-100_F4 | ACTCTGAAACCAGTGGGAATGT |
| SOX-115-100_IF4 | TCACATAGAACAGAGCCCAGTG |
| SOX-115-100_F5 | TGTTTGCCCATCTGTCTATCTG |
| SOX-115-100_IF5 | GCATTCACACGTGATGTCAAAC |
| SOX-115-100_F6 | AACAAGACTGCATGGAGATGAA |
| SOX-115-100_F7 | CTGGGGTTTAGCATTCTGTTTC |
| SOX-115-100_IR1 | GTTTGGCTTTGCAATTTGCTT |
| SOX-115-100_R1 | ATTCATTAGATACGATGGGCCAAA |
| SOX-115-100_IR2 | GCAGGTTCAGTGAAATCTCAGG |
| SOX-115-100_R2 | GTGCAGTCAGTGGGTATACTGG |
| SOX-115-100_IR3 | CCCCCTCAGCTGTATATCAAGT |
| SOX-115-100_R3 | TAGGGTTTGGACAGTCTGTGTG |
| SOX-115-100_IR4 | CCTCAAACATAGCAACACCATT |
| SOX-115-100_R4 | TGTCTCTTTAAATGGGAACACC |
| SOX-115-100_IR5 | GGCCTGTTAAACTGCTTCTGG |
| SOX-115-100_R5 | CAGGAATGCGACTTCTTCCCTA |
| SOX-115-100_R6 | GTATGACCCTCCTACGCCTTTT |
| SOX-115-100_R7 | CCAAACCAAAACAAAATCTTCC |
| SOX-115-100_IR7 | ATTGAACACGACAGGCAGGC |
| SOX-115-100 7_IF | TGGACATCTGTTTAACAATT |
| SOX-115-100 7_IR | AATCAGCATTTTATTCATTTT |
|  |  |
| **Gap coverage and Dyna-bead primers** | |
| LR_gap1_68001735_F1 | CTATTCAGAGCCCCTCCTAAGCAAA |
| LR_gap1_68002038_F2 | CTCCTCTCCGTTGACATTTCCTGT |
| LR_gap1_68000780_R1 | ATCTCCAGAGAAGGAGACACGACAA |
| LR_gap1_68005179_R2 | TGAGCTGCTGACTGTAGATGTTTGG |
| Dynal_-75_gap_F1_bio | GCAGTATCAAAACGCTTCCTTG |
| Dynal_-75_gap_F2_bio | CGAACTGTATTGCTGGACCTTC |
| Dynal_-75_gap_F2 | CGAACTGTATTGCTGGACCTTC |
| Dynal_-75_gap_F3 | TGTGAGGGAGGTATGCCTTCTA |
| Dynal_-75_gap_R1_bio | GCAGAGGAGCTCAAAGAACCAT |
| Dynal_-75_gap_R2_bio | CTTATGTCCAAGAGGGGAGAGC |
| Dynal_-75_gap_R2 | CTTATGTCCAAGAGGGGAGAGC |
| Dynal-105_gap_F1_bio | CACTGTTCAAAAGGCGTAGGAG |
| Dynal-105_gap_F2_bio | CGTAGGAGGGTCATACCCAAT |
| Dynal-105_gap_F2 | CGTAGGAGGGTCATACCCAAT |
| Dynal-105_gap_F3 | GGGTCATACCCAATTTTGATGT |
| Dynal-105_gap_R1_bio | TTTCAAGTTTCAACAAGGACCA |
| Dynal-105_gap_R2_bio | GTCTCCACATGAAACAGAATGC |
| Dynal-105_gap_R2 | GTCTCCACATGAAACAGAATGC |
| Dynal-105_gap_R3 | CTCCCAATCCTGCTTTACATTT |
| SOX-80_gap_IR2_68003655 | CCCTACAGCCATCACCTGTAA |
| SOX-80_gap_IF2_68002125 | AGCCACAGAGCTGTCCTCCT |
| DYNAL-SOX5_ex1_F1_bio | GGGCTCGGGCTCACTTGACAGGTTCA |
| DYNAL-SOX5_ex1_F2 | GGTGGCTGCTGTGACAAAGGGAAACT |
| DYNAL-SOX5_ex1_R1_bio | AGTGAGCATGCTGGAAAAGCGCAGTT |
| DYNAL-SOX5_ex1_R2 | TGGTCGCCTCCGACTGAACCTGTCAA |
| SOX-75_gap-bridge_F | ACCCAAGAAGTCTCCTCTCCTC |
| SOX-75_gap-bridge_R | AATGACTCAAATACAGGCCACA |
| gap_9k_F | CAGTGGGAGTTCAGAATGACAG |
| gap_9k_R | CTTCCAAGCTATTTTGTTGCAC |
| gap_10k_F | TCTGCACTTTAAAACCTTTCAGG |
| gap_10k_IR | TCCCATTTGACTGCCTCTTAAT |
| gap_10k_R | GCTCAGCCTCAATAGGAGAAAA |
| gap_11.9kIF | TGGACATCTGTTTAACAATT |
| gap_11.9kIR | CAGCATTTTATTCATTTTGAT |
| Gap_16880_F | TGAGATCAGAGAGGAGGCTAGG |
| Gap_16880_R | TGGGGCTTTATAAGACATCACA |
| Gap_24900_F | CTCGGAGCTCTAATGGCTTAAA |
| Gap_24900_R | TTCAGTGTGAGCAAACTCTGGT |
| Gap_24900_F_internal | TTGATTTAAACCATTCCTCCCATC |
| Gap_24900_R_internal | GTGTGAGCAAACTCTGGTGTGT |
| Gap_12000-13300_F | CACGCACACACACACACAA |
| Gap_12000-13300_R | TCAGTGCCTTGTCATCCAAA |
| Gap_12000-13300_IF1 | GAACACTGTTGCCTGCCTGT |
| Gap_12000-13300_IR1 | AATCAGCATTTTATTCATTTTGATTG |
| Gap_12000-13300_IF2 | CGACGAGGAGGGGGAATC |
| Gap_12000-13300_IR2 | TGGTAACCATGTGTGGCAAG |
| Gap_12000-13300_IF3 | CCCTTCTCCCCTGTTTCTG |
|  |  |
|  |  |
| **Duplication sequencing primers** | |
| SOX-Duplication_LR1_R | CCCTGTGGCAGCTTCTGAATATCT |
| SOX-Duplication_LR1_F | CTATTCAGAGCCCCTCCTAAGCAAA |
| SOX-Duplication_LR2_F | TCTCTGCAGACCAGAGTGTGTCTGC |
| SOX-Duplication_LR2_R | CACATCCCCATGGAACACGAGGAGC |
| SOX-Duplication_LR3_F | GAATACTTCGCATTTGTCCACCTTG |
| SOX-Duplication_68000100_F11 | CAGCTCCCCTTGTTCCATTGATTAC |
| SOX-Duplication_68000100_R11 | CAATGGGGTAATCAATGGAACAAGG |
| SOX-Duplication_67999400_F12 | AGAGATGGTGAAATCCAGGTCGTC |
| SOX-Duplication_67999400_R12 | GAGGACGACCTGGATTTCACCAT |
| SOX-Duplication_67998700_F13 | GCAGAGAGACCTGCTGCTGCT |
| SOX-Duplication_67998700_R13 | GAGCCCCACAAGGAGATGGAG |
|  |  |
|  |  |
| **5' RACE and cDNA Primers** | |
| SOX5CDNA_1_F | AGGAACAGATTGCAAGACAACA |
| SOX5CDNA_1_R | CTGCTGGATCTGTTGCTGAAG |
| SOX5_CDNA_5'RACE_F1 | ACAGGTTCAGTCGGAGGCTA |
| SOX5_CDNA_5'RACE_R1 | TTGTCACAGACGCCACCTTA |
| SOX5_CDNA_5'RACE_R2 | GTCAGTGAGCATGCTGGAAA |
| SOX5_CDNA_5'RACE(2)_R1 | CTCCATCTGCTTCCCCATAC |
| SOX_cDNA_ex2_F | GATGTCTTCCAAGCGACCAG |
| SOX_cDNA_ex2_R | GCCCGTCACTCTCCTCTTCT |
| SOX_cDNA_ex3_F | TTGATGGCAATAAAGCGATG |
| SOX_cDNA_ex3_R | GCTGTGCTGGACAATGATTC |
| SOX_cDNA_ex4_F | CAAAGGACTGGAAAGACAAGC |
| SOX_cDNA_ex4_R | TCTCCAAAGTTCCCTGATCC |
| SOX_cDNA_ex5_F | CTGACCAGTTTGCGAGAACA |
| SOX_cDNA_ex5_R | GTTCCTGTTGCTGCTTAGCC |
| SOX_cDNA_ex6_F | ATTGCAAGACAACAGCAGCA |
| SOX_cDNA_ex6_R | TGGATCTGTTGCTGAAGAAGG |
| SOX_cDNA_ex7_F | CTCCAGACCAACGAACCCTA |
| SOX_cDNA_ex7_R | TCCCGCTTTGTAGCTGAAAC |
| SOX_cDNA_ex8_F | CAGCTGATCCCGACTACCAT |
| SOX_cDNA_ex8_R | CCTAGGCCTGGTGTTGCTG |
| SOX_cDNA_ex9_F | TCCAGGAGGCAAGATACCTG |
| SOX_cDNA_ex9_R | AGGGCTGTTTGTGCTCTTGT |
| SOX_cDNA_ex10_F | AGCCAAACCCAAGACATCTG |
| SOX_cDNA_ex10_R | TAGCTCTGGCCGAAGGACTA |
| SOX_cDNA_ex11_F | CCATGATGCTGTTACCAAGG |
| SOX_cDNA_ex11_R | ATTCACAACAGCCACCTTCC |
| SOX_cDNA_ex12_F | ACGACACTGGAGACCCTGAC |
| SOX_cDNA_ex12_R | TCAGAATCTCCACTCATGTTGAA |
| SOX_cDNA_ex13_F | AATTTATCGGGAATCCAGAGG |
| SOX_cDNA_ex13_R | AGGGAAGGCTTGAAGGATCT |
| SOX_cDNA_ex14_F | CAGCACCTGGAGAAGTACCC |
| SOX_cDNA_ex14_R | ACGTCTGTTGCGCATGATAG |
| SOX_cDNA_ex15_F | TTGTTTACCCAGGAGCCATC |
| SOX_cDNA_ex15_R | TTGATGCCGTAAGTGCTCTG |
| ß -actin_F | AGGTCATCACCATTGGCAATG |
| ß -actin_R | CCCAAGAAAGATGGCTGGAA |
| Tata Box BP_F | TAGCCCGATGATGCCGTAT |
| Tata Box BP_R | GTTCCCTGTGTCGCTTGC |
|  |  |
| **Southern Blot Primers** |  |
| SOX-55kb_SB_F | GGCTCTGAGCAGGTGTAGTAGG |
| SOX-55kb_SB_R | AAGTATTTCTTTGGGGCAGTGA |
| SOX-65kb_SB_F | ACAGGGCGGGTTTTTAGTATTC |
| SOX-65kb_SB_R | AAGAGGAAGAAGAATGGACGTG |
| SOX-75kb_SB_F | ATGTGGGCACACTGTAAGACTG |
| SOX-75kb_SB_R | AAACACCACCGAATGTTCTTCT |
| SOX-85kb_SB_F | GAGATATTCAGAAGCTGCCACA |
| SOX-85kb_SB_R | GCAGGGATTGCACTTAAATGAT |
| SOX-95kb_SB_F | TGTCATCCTGAGGGATTTACAA |
| SOX-95kb_SB_R | TTTCACAAGGTGATCAGAACTCTC |
| SOX-105kb_SB_F | TCACATAGAACAGAGCCCAGTG |
| SOX-105kb_SB_R | CCTCAAACATAGCAACACCATT |
