# Supplementary material for: Copy Number Variation in Intron 1 of SOX5 Causes the Pea-comb Phenotype in Chickens
Source: PLoS Genet. 2009 Jun 12;5(6):e1000512. doi: 10.1371/journal.pgen.1000512 (PMC2685452; doi:10.1371/journal.pgen.1000512)
Supplement: Table S2 — Primers and TaqMan probes for real-time PCR analysis of chicken SOX5. (0.03 MB DOC) [file pgen.1000512.s004.doc]

**Supplementary Table S2**. Primers and TaqMan probes for real-time PCR analysis of chicken *SOX5*.

| Primer Name | Sequence (5’ – 3’) |
| --- | --- |
| BamH1_pcomb-forward | GGGCTCCTCGTGTTCCAT |
| BamH1_pcomb-reverse | CCACCGGAAATGTCAACAGAGA |
| BamH1_pcomb-probe | CAGAGGCTGTCCAACATG |
| rps24-forward | gggaagtaaacatcagtcgacaaa |
| rps24-reverse | tttgccaagccttgcatct |
| rps24-probe | caatcaatcttcaccgctgcttccca |
