# Supplementary material for: Copy Number Variation in Intron 1 of SOX5 Causes the Pea-comb Phenotype in Chickens
Source: PLoS Genet. 2009 Jun 12;5(6):e1000512. doi: 10.1371/journal.pgen.1000512 (PMC2685452; doi:10.1371/journal.pgen.1000512)
Supplement: Table S3 — Results of real-time PCR analysis of the duplicated fragment in single comb and Pea-comb chickens. (0.08 MB DOC) [file pgen.1000512.s005.doc]

**Supplementary Table S3.** Results of real-time PCR analysis of the duplicated fragment in single comb and Pea-comb chickens.

| Population | Sample name | Fold^a^ | Range^b^ | |
| --- | --- | --- | --- | --- |
| Red junglefowl | 448 | 1.4 | 1.0 | 2.0 |
|  | 239 | 0.7 | 0.6 | 0.8 |
|  | 240 | 0.4 | 0.4 | 0.4 |
|  | 311 | 0.6 | 0.5 | 0.6 |
|  | 321 | 0.2 | 0.2 | 0.2 |
| Broiler | 5 | 0.3 | 0.2 | 0.4 |
|  | 6 | 0.3 | 0.2 | 0.4 |
|  | 2 | 0.1 | 0.1 | 0.2 |
|  | 12 | 0.4 | 0.4 | 0.5 |
|  | 15 | 0.6 | 0.5 | 0.7 |
| Czech Golden Pencilled | 1P24 | 5.3 | 4.3 | 6.7 |
|  | 2P24 | 1.0 | 0.8 | 1.1 |
|  | 3P24 | 3.0 | 2.6 | 3.4 |
|  | 4P24 | 0.3 | 0.2 | 0.3 |
|  | 5P24 | 2.8 | 2.5 | 3.2 |
| Friesian Fowl | 2B1001-02-P10 | 0.2 | 0.2 | 0.3 |
|  | 2B1001-03-P10 | 0.2 | 0.1 | 0.2 |
|  | 2B1001-06-P10 | 0.1 | 0.0 | 0.1 |
|  | 2B1001-07-P10 | 0.0 | 0.0 | 0.1 |
|  | 2B1001-08-P10 | 0.1 | 0.1 | 0.1 |
| Finnish Landrace | 7P17 | 0.1 | 0.1 | 0.2 |
|  | 8P17 | 0.1 | 0.0 | 0.1 |
|  | 10P17 | 0.2 | 0.2 | 0.2 |
|  | 12P17 | 0.2 | 0.2 | 0.3 |
|  | 9P17 | 0.3 | 0.3 | 0.4 |
| Red Villafranquina | 1P23 | 0.1 | 0.1 | 0.1 |
|  | 3P23 | 0.0 | 0.0 | 0.0 |
|  | 26P23 | 0.2 | 0.1 | 0.2 |
|  | 6P23 | 0.3 | 0.2 | 0.4 |
|  | 7P23 | 0.2 | 0.2 | 0.3 |
| Transylvania Naked Neck | 1P26 | 0.1 | 0.0 | 0.1 |
|  | 2P26 | 0.6 | 0.5 | 0.7 |
|  | 3P26 | 0.2 | 0.2 | 0.2 |
|  | 4P26 | 1.0 | 0.9 | 1.0 |
|  | 5P26 | 1.2 | 1.1 | 1.2 |
| White Leghorn | 303 | 0.2 | 0.1 | 0.3 |
|  | 323 | 0.2 | 0.1 | 0.2 |
|  | 301 | 0.2 | 0.2 | 0.3 |
|  | 352 | 0.4 | 0.2 | 0.8 |
|  | 366 | 0.2 | 0.2 | 0.2 |
| French Pea-comb | 6430 | 24.2 | 22.4 | 26.2 |
|  | 6458 | 28.0 | 22.2 | 35.4 |
|  | 6476 | 8.6 | 6.6 | 11.2 |
| Hua-Tung (Pea-comb) | 212211 | 23.8 | 22.7 | 25.0 |
|  | 216152 | 0.3 | 0.3 | 0.3 |
|  | 217132 | 11.9 | 11.5 | 12.3 |
|  | 220052 | 12.1 | 11.4 | 12.9 |
|  | 220212 | 6.3 | 6.0 | 6.5 |
|  | 221172 | 13.9 | 12.8 | 15.1 |
|  | 223342 | 10.3 | 9.5 | 11.1 |
|  | 182551 | 14.0 | 12.0 | 16.2 |
|  | 212931 | 23.1 | 22.0 | 24.2 |
|  | 213171 | 18.4 | 17.4 | 19.3 |
|  | 216171 | 21.6 | 19.8 | 23.5 |
|  | 221901 | 12.6 | 12.2 | 13.0 |
|  | 223601 | 15.0 | 13.4 | 16.9 |
| Orlov (Pea-comb) | 1091 | 26.7 | 25.8 | 27.6 |
|  | 1131 | 27.5 | 25.4 | 29.7 |
|  | 1141 | 14.8 | 14.3 | 15.4 |
|  | 1282 | 20.8 | 19.3 | 22.3 |
|  | 1302 | 17.9 | 17.2 | 18.7 |
|  | 1332 | 15.7 | 15.0 | 16.4 |
|  | 1081 | 45.2 | 42.3 | 48.4 |
|  | 1101 | 33.2 | 31.1 | 35.4 |
|  | 1272 | 22.0 | 20.9 | 23.2 |
|  | 1322 | 31.6 | 30.2 | 33.1 |
|  | 1342 | 18.0 | 16.7 | 19.3 |
|  | 1352 | 32.3 | 30.4 | 34.3 |
|  |  |  |  |  |

^a^Fold change was calculated using the equation 2^-(Normalised Ct Pea-comb assay – Normalised Ct rps24 assay). ^b^Fold change range was determined from the combined standard errors of both assays. All assays were performed in triplicate.
